# A Mouse Model of Ulcerative Cutaneous Leishmaniasis by *Leishmania (Viannia) panamensis* to Investigate Infection, Pathogenesis, Immunity, and Therapeutics

**DOI:** 10.3389/fmicb.2022.907631

**Published:** 2022-06-13

**Authors:** Natalia Muñoz-Durango, Alexander Gómez, Natalia García-Valencia, Miguel Roldán, Marcela Ochoa, David E. Bautista-Erazo, José R. Ramírez-Pineda

**Affiliations:** ^1^Grupo Inmunomodulación (GIM), Instituto de Investigaciones Médicas, Facultad de Medicina, Corporación Académica para el Estudio de Patologías Tropicales (CAEPT), Universidad de Antioquia, Medellín, Colombia; ^2^Instituto de Patología, Facultad de Medicina, Universidad de Antioquia, Medellín, Colombia; ^3^Programa de Estudio y Control de Enfermedades Tropicales (PECET), Facultad de Medicina, Universidad de Antioquia, Medellín, Colombia

**Keywords:** cutaneous leishmaniasis, *Leishmania (Viannia) panamensis*, mouse model, vaccine, immunopathology, forward vaccinology

## Abstract

A mouse model of cutaneous leishmaniasis (CL) by *Leishmania (Viannia) panamensis (L(V)p)* that reproduces the characteristics of the human disease remains elusive. Here we report the development of a CL model that uses a mouse-adapted *L(V)p* isolate to reproducibly induce a dermal disease with a remarkable similarity to human CL. BALB/c mice infected intradermally in the ear with 10^5^ stationary UA-946 *L(V)p* promastigotes develop a progressive cutaneous disease that exhibits the typical ulcerated lesions with indurated borders observed in CL patients. Although most of parasites in the inoculum die within the first week of infection, the survivors vigorously multiply at the infection site during the following weeks, paralleling disease appearance and aggravation. Regional lymphadenopathy as well as lymphatic dissemination of parasites to draining lymph nodes (dLN) was evidenced early after infection. Viable parasites were also isolated from spleen at later timepoints indicating systemic parasitic dissemination, but, strikingly, no signs of systemic disease were observed. Increasing numbers of myeloid cells and T lymphocytes producing IFNγ and IL-4 were observed in the dLN as disease progressed. A mixed adaptive *L(V)p*-specific T cell-mediated response was induced, since *ex vivo* recall experiments using dLN cells and splenocytes revealed the production of type 1 (IFNγ, IL-2), type 2 (IL-4, IL-13), regulatory (IL-10), and inflammatory (GM-CSF, IL-3) cytokines. Humoral adaptive response was characterized by early production of IgG1- followed by IgG2a-type of *L(V)p*-specific antibodies. IFNγ/IL-4 and IgG2a/IgG1 ratios indicated that the initial non-protective Th2 response was redirected toward a protective Th1 response. *In situ* studies revealed a profuse recruitment of myeloid cells and of IFNγ- and IL-4-producing T lymphocytes to the site of infection, and the typical histopathological changes induced by dermotropic *Leishmania* species. Evidence that this model is suitable to investigate pharmacological and immunomodulatory interventions, as well as for antigen discovery and vaccine development, is also presented. Altogether, these results support the validity and utility of this novel mouse model to study the pathogenesis, immunity, and therapeutics of *L(V)p* infections.

## Introduction

Mouse models have been extremely useful to dissect the immune mechanisms mediating leishmaniasis pathogenesis and host resistance to *Leishmania* parasites, as prototypic intracellular microorganisms. Inbred mouse strains have been infected with different *Leishmania* species, and a variety of outcomes, ranging from asymptomatic infection to fatal systemic disease, have been observed. The resulting picture of the reported experimental work is that whereas some pathways are used by the host immune system to deal with all tested *Leishmania* species, other pathways operate in a species-specific manner. For instance, whereas a sustained Th1 IFNγ-mediated response is required for protection in all tested models, a non-healing disease might be the result of a dominant Th2 response, a weak Th1 response, a prominent regulatory response (e.g., IL-10- or TGFβ-mediated) or even from B cell responses, depending on the species involved (McMahon-Pratt and Alexander, 2004). Innate immune cells and pathways are also differently modulated by *Leishmania* species, contributing to disease or protection in a different manner (Hurrell et al., 2016; Zamboni and Sacks, 2019). Moreover, host response to *Leishmania* can further diverge at the strain level within the same species, as indicated by the inflammasome/neutrophil-mediated non-healing response observed with the Seidman strain of *Leishmania (Leishmania) major* (*L(L)m*) even in the presence of a robust Th1 response (Charmoy et al., 2016). Conversely, many virulence factors that have been identified in some species (e.g., the lipophosphoglycan, the A2 protein, or cysteine protease enzymes) are not expressed or are non-operative in other (McMahon-Pratt and Alexander, 2004; Rossi and Fasel, 2018a). This species-specific nature of the mammalian host–*Leishmania* interaction implies that information gained with a particular species cannot be assumed to apply across the whole genus or subgenus.

*Leishmania (Viannia) panamensis (L(V)p)* is one of the most important causal agents of American cutaneous leishmaniasis (CL) and is responsible for a large proportion of leishmaniasis cases in Central and South America (Alvar et al., 2012; Aronson et al., 2020). *L(V)p* has been isolated from patients with different manifestations of the disease, including the localized (LCL), mucosal (ML), and disseminated (DisCL) forms (World Health Organization, 2010; Vélez et al., 2015; Scorza et al., 2017). Clinical–epidemiological studies have revealed intriguing characteristics of the natural history of *L(V)p* infection in endemic regions, such as the variable frequency of asymptomatic infections and spontaneous healing, latency, evolution to chronic disease and/or recurrence, and metastatic behavior (Weigle and Saravia, 1996; World Health Organization, 2010). It is generally assumed that this broad spectrum of outcomes upon infection is the result of the interactions between parasite- and host-derived factors in a particular environment.

Syrian hamsters have been claimed as a suitable animal model to investigate host response to *L(V)p* and other *Leishmania* species of the *Viannia* subgenus, since persistent cutaneous lesions are induced upon promastigote inoculation in those animals (Hommel et al., 1995; Osorio et al., 2003). Interestingly, as observed in humans, parasites persist for long periods and disseminate not only to draining lymph nodes (dLN) but also to distant tissues where metastatic lesions are observed (Travi et al., 1988; Saravia et al., 1990; Martinez et al., 1991). Cytokine mRNA measurements in the site of infection, dLN, and spleen evidenced the expression of IFNγ, IL-10, IL-4, IL-12, and TGFβ (Travi et al., 2002; Osorio et al., 2003; Espitia et al., 2010), which resembles the mixed Th1/Th2/regulatory profile observed in humans (Bosque et al., 2000; Trujillo et al., 2002; Castilho et al., 2010; Díaz et al., 2010). Whereas this model has been useful to investigate mechanisms of parasite virulence (Acestor et al., 2006), the unavailability of genetically homogeneous inbred strains, together with the scarcity of immunologic/molecular tools and genetic manipulation systems in hamsters, has limited its use to map the host immune pathways that are relevant for pathogenesis or immunity. Although murine models would be convenient for this purpose, species of *Viannia* subgenus were early recognized as poorly infective in mice (Hommel et al., 1995; Travi et al., 2002; McMahon-Pratt and Alexander, 2004), probably due to the difficulties to reproducibly induce measurable and sustained cutaneous lesions in these animals. Even in BALB/c mice, where most of mammalian-infecting *Leishmania* species can grow leading to overt disease, *Leishmania (Viannia) guyanensis* (*L(V)g*) and *Leishmania (Viannia) braziliensis* (*L(V)b*) infection are generally asymptomatic or induce a mild disease that resolves within few weeks with almost complete elimination of parasites (de Moura et al., 2005; Sousa-Franco et al., 2006; de Oliveira and Brodskyn, 2012). Although McMahon-Pratt’s group reported the injection of putative infective forms in the foot of BALB/c mice, the resulting disease consists of small non-ulcerative and poorly characterized lesions (Castilho et al., 2010), which appear very limited to model the tissue-damaging characteristics of human CL by *L(V)p*. Thus, to our knowledge, a suitable murine model of CL by *L(V)p* that mimics the characteristics of the human disease has not been developed and characterized. Here, we report a clinical isolate of *L(V)p* able to reproducibly induce chronic ulcerative lesions upon intradermal (id) injection in the ear of BALB/c mice that closely resembles the human disease caused by this species. The kinetics of clinical, parasitological, immunological, and histopathological response to the infection was systematically investigated, and the resulting model was used to evaluate the efficacy of pharmacological and immunological interventions. Our findings demonstrate that BALB/c mice are susceptible to infection with *L(V)p*, providing a suitable new model to study the pathogenesis and immunity to *Leishmania* parasites and to assess drug and vaccine candidates.

## Materials and Methods

BALB/c mice (Charles River, United States) were used in all experiments, and procedures were approved by the institutional ethical animal committee. From the twelve clinical *L(V)p* isolates tested in mice (Supplementary Table S1), the UA-946 stock (MHOM/CO/93/UA-946) was chosen for the establishment of the model. The genome sequence of the UA-946 *L(V)p* isolate has been reported (Urrea et al., 2018). *L(L)m* (MHOM/IL/81/BNI; Ramírez-Pineda et al., 2004) was used in some experiments for comparison. Total *Leishmania* antigen (Ag) was prepared by freezing/thawing cycles, and fractionation methods were used for some procedures. Animals were infected into the right hind footpad, the base of the tail, or the right ear, subcutaneously (sc) or id, with stationary promastigotes, and a clinical follow-up was performed consisting in the assessment of body weight and lesion development. The footpad thickness and lesion size were registered weekly with the help of a digital caliper (precision: 0.02 mm, model MT-00855, Uyustools, China). The footpad swelling was calculated as the difference in the thickness (in mm) between the infected and the contralateral non-infected foot. Lesion size in the base of the tail and the ear was reported as the area (in mm^2^) by measuring the two crossed diameters of lesions and calculating with the formula $A=\pi {\left(\frac{D1+D2}{4}\right)}^{2}$. At different timepoints after infection, mice were euthanized to obtain tissues for parasitological, histopathological, and immunological analysis. Infected tissues were removed, weighted, and cultured to determine the parasite burden per organ (footpad and ear) or mg of tissue (base of the tail) by using a limiting dilution protocol (Buffet et al., 1995; Lima et al., 1997). Draining LNs and spleens were weighted and cell suspensions seeded in promastigote culture medium and incubated at 26^o^C to determine the presence of viable parasites. In chemotherapeutic experiments, mice received treatment: a weekly intraperitoneal (ip) injection of Glucantime^®^, 500 mg/kg, for 4 weeks, or Miltefosine, 20 mg/kg/day, orally for five consecutive days, administered after disease establishment (4–5 weeks postinfection). In immunomodulatory interventions, the synthetic CpG-containing oligodeoxynucleotide 1826 (CpG; 5′-TCCATGACGTTCCTGACGTT-3′; phosphorothioate-modified; Integrated DNA Technologies - IDT, United States) was used for co-delivery or as a vaccine adjuvant with total Ag or protein fractions. Schemes, doses, and administration routes employed for each independent experiment are specified in the corresponding figure legend. Cytokine concentrations in supernatants of dLN or spleen cells stimulated with 10 μg *L(V)p* Ag for 72 h were determined by ELISA and/or Luminex. *L(V)p*-specific IgG1 and IgG2a antibodies in serum were quantified by ELISA. Flow cytometry of cell suspensions from infected tissue and dLN was performed as reported (Duthie et al., 2007; Anderson et al., 2008) and myeloid and T cells analyzed. For further details on methodology and data analysis, see Supplementary Materials and Methods.

## Results

### Selection of a Suitable *L(V)p* Human Isolate for BALB/c Mouse Infections

Although our previous efforts to induce a cutaneous disease in mice with *L(V)p* were disappointing, the finding of viable parasites and a robust IgG *L(V)p*-specific Ab response in chronically infected asymptomatic animals (not shown) indicated that *L(V)p* was able to infect, survive, and persist in mouse tissues and to elicit a parasite-specific adaptive response. On the bases of the significant heterogeneity exhibited by field isolates of the same *Leishmania* species on mouse pathogenicity (Monroy-Ostria et al., 1994; Barral et al., 1996; de Oliveira and Brodskyn, 2012; Loeuillet et al., 2016) and the reports using serial passages *in vivo* to adapt microbial pathogens to grow in mouse tissues and produce successful infections (Marchetti et al., 1995; Xu et al., 2011; Li and McCray, 2020), we implemented a two-step strategy of selection-adaptation. The strategy consisted in the initial identification of clinical isolates that better infected mouse tissues followed by a second step of *in vivo* adaptation (as described in Supplementary Materials and Methods). Thus, we tested 12 human isolates representing different clinical forms of leishmaniasis caused by *L(V)p* (localized, mucosal, disseminated, or recurrent cutaneous leishmaniasis; LCL, ML, DisCL or RCL, respectively; Supplementary Table S1), and although most of the *L(V)p* infections were asymptomatic, three isolates produced mild symptomatic infections in the footpad of BALB/c mice (Supplementary Figure S1). As reported for other species (Monroy-Ostria et al., 1994; Alves-Ferreira et al., 2015; Loeuillet et al., 2016), there was no relation between human and mouse pathogenicity/virulence. As expected, parasites could be cultured from infected tissue and the dLN of animals infected with the three pathogenic isolates 3 or 8 months after infection (Supplementary Table S2). This indicated that some human *L(V)p* isolates successfully infect BALB/c mice, provoking a measurable inflammation in the footpad. One isolate, coded UA-946 and exhibiting the most consistent results in independent experiments using several routes/sites, was selected for further *in vivo* adaptation *via* serial passages in BALB/c mice and for subsequent characterization.

### Localized Non-ulcerative Chronic Lesions in the Footpads of Mice Infected With *L(V)p*

**Figure 1:**
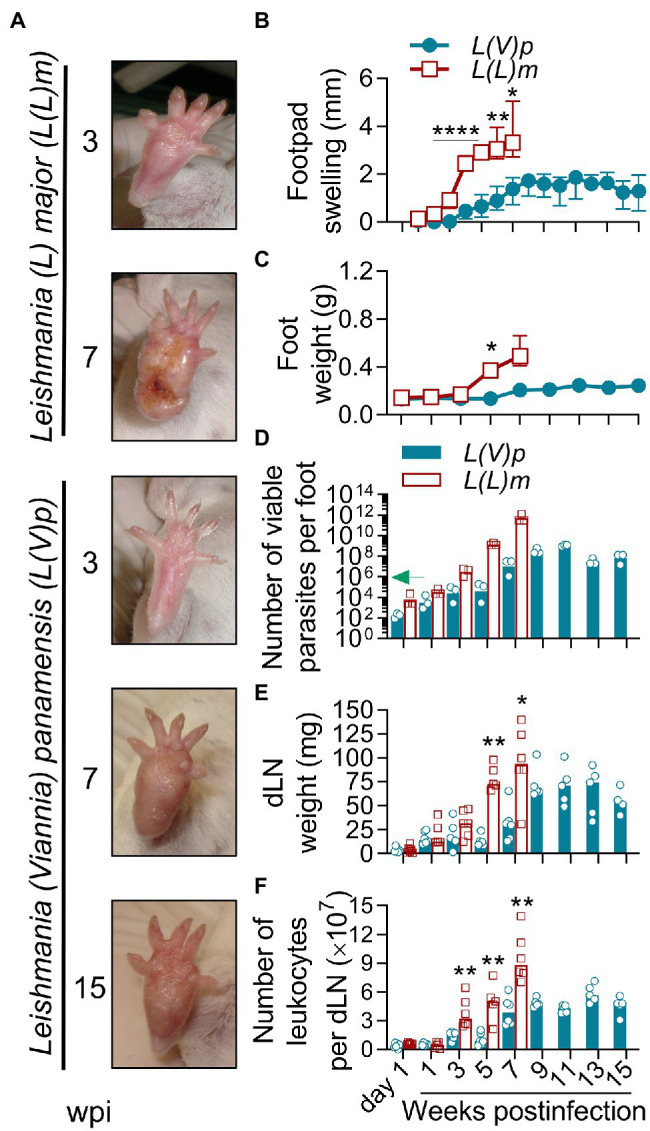
Localized non-ulcerative chronic lesions in the footpads of mice infected with *L(V)p*. BALB/c mice were infected subcutaneously (sc) with 10^6^ *Leishmania (Viannia) panamensis* (*L(V)p*) stationary promastigotes in the right footpad and monitored weekly. In parallel, a group of mice were infected with a similar inoculum of *Leishmania (Leishmania) major* (*L(L)m*) promastigotes as a control with a highly pathogenic *Leishmania* species in BALB/c mice. Representative photographs at the indicated time postinfection are presented **(A)**. The severity of lesions in mice infected with *L(L)m* at the 7th week postinfection obligated immediate euthanasia. Footpad swelling was individually measured and graphed **(B)**. At the indicated time, mice were sacrificed and the weight **(C)** and the number of viable parasites **(D)** of the foot determined. Draining lymph nodes (dLN) were also removed and the weight **(E)** and cellularity **(F)** determined. Results are expressed as the median ± interquartile range **(B)**, median ± range **(C)**, geometric mean **(D)** or median **(E,F)** from 2–7 mice per time/group. The arrow in **(D)** indicates the infective inoculum. wpi, weeks postinfection. ^*^p < 0.05, ^**^p < 0.01, and ^****^p < 0.0001 [mixed-effects analysis **(B)**; two-way ANOVA **(C)** with Geisser–Greenhouse correction and Bonferroni’s multiple comparisons *post-hoc* test for multiple comparison through time; Mann–Whitney *U*-test **(E,F)**].

We first evaluated the kinetics of lesion development and parasite replication after UA-946 *L(V)p* inoculation in the footpad of BALB/c mice and compared it to that of *L(L)m*, a well-documented model of leishmaniasis susceptibility. Although infection with *L(V)p* was asymptomatic during the first 3 weeks, it progressed to a nodular non-ulcerated inflammatory lesion that reached maximal size around 8–9 weeks postinfection (Figures 1A,B). No further aggravation of the inflammatory lesions was observed throughout the study, instead footpad swelling tended to reduce toward weeks 14 and 15 postinfection (Figure 1B). Mice looked healthy, and body weight and behavior were comparable to non-infected animals. In contrast and as expected, mice infected with *L(L)m* exhibited footpad inflammation as early as 2 weeks postinfection that rapidly progressed to ulcers and aggravated to necrosis by the 7th week postinfection (Figures 1A,B). At this time, *L(L)m*-infected mice displayed weight loss and symptoms of systemic disease (hypoactivity and piloerection), forcing humane sacrifice. Foot weight measurement confirmed that whereas both species promoted a progressive inflammatory process in BALB/c mice, inflammation induced by *L(V)p* was slower and limited (Figure 1C). From the 10^6^ *L(V)p* parasites injected into BALB/c mice only hundreds could be recovered 1 day postinfection; however, this surviving fraction was able to actively multiply within the following 11 weeks in a 6-log factor (Figure 1D). Interestingly, for the remaining observation period, viable parasite loads stabilized indicating an effective control of parasite replication by the host during the chronic phase. From a similar inoculum of *L(L)m*, a bigger fraction (thousands) survived the first day, and surviving parasites rapidly replicated (8-log factor) to reach levels of hundreds of billions within few weeks. These results indicate that *L(V)p* provokes in the footpads of BALB/c mice a non-ulcerative, self-limited, and localized chronic lesion that contrasts with the severe, systemic, and potentially lethal disease caused by *L(L)m* in these mice.

### Infection With *L(V)p* in the Ear Dermis Induces Chronic Ulcers That Closely Resemble Human CL

The confirmation of active parasite replication in the footpads of mice infected with *L(V)p* prompted us to examine the response to infection in other anatomical locations. In pilot experiments, we found that injection of 10^5^ parasites in the base of the tail or the ear was sufficient to induce lesions within 4 weeks postinfection (data not shown). Therefore, this inoculum was chosen for further experiments. BALB/c mice infected sc in the base of the tail exhibited an early nodular small lesion that progressed to either larger nodules or ulcerative lesions within the following weeks postinfection (Supplementary Figures S2A,B). Although some animals developed typical ulcers, other never developed ulcerated lesions. Among different experiments, lesions (either nodules or ulcers) tended to be heterogeneous in size and appearance and less reproducible (Supplementary Figures S2A–D). The kinetics of parasitic load at the infection site, again, evidenced the presence of few viable parasites at the asymptomatic early phase, followed by an active phase of parasite multiplication, which coincides with the onset of disease, and then by a late chronic phase where the control of parasite replication was observed (Supplementary Figure S2E).

**Figure 2:**
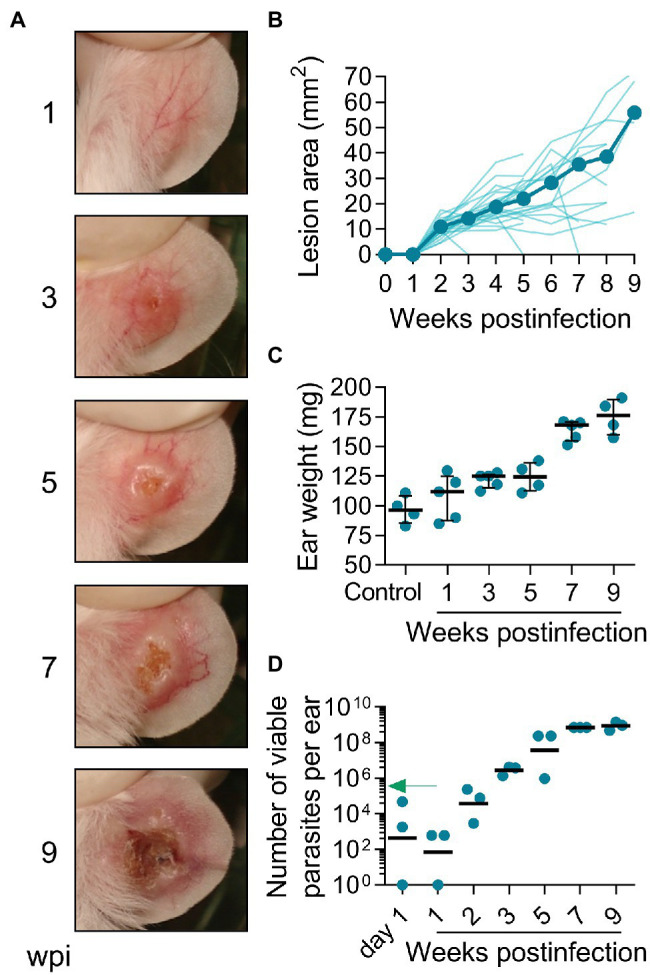
Infection with *L(V)p* in the ear dermis induces chronic ulcers that closely resemble human CL. BALB/c mice were infected with 10^5^ *L(V)p* stationary promastigotes id in the right ear and monitored weekly. Representative photographs at the indicated time postinfection are presented **(A)**. The size of the lesion was measured and graphed as the median [thick line] and for each individual mouse [thin lines] **(B)**. At the indicated time, mice were sacrificed, and the ears removed to measure the weight **(C)** as an indicator of inflammation. Results are presented as the median ± interquartile range in **(C)**. The number of viable parasites **(D)** in the ears was also determined by limiting dilution and graphed, with lines representing the geometric mean. The arrow in **(D)** indicates the infective inoculum. *n* = 3–5 mice per time. wpi: weeks postinfection.

In humans, *L(V)p* promastigotes are deposited in the dermis during sandfly biting, and after an incubation period that ranges from 2 weeks to several months, the infiltration of immune cells leads to the formation of a small papule that progresses to form the typical ulcerated lesion that characterizes LCL by *L(V)* species. Although spontaneous healing is observed in a significant proportion of CL patients, skin lesions might last from months to years (Weigle and Saravia, 1996). We therefore switched to a more physiological route of infection by injecting BALB/c mice id in the ear with UA-946 *L(V)p* promastigotes and monitoring the animals clinically and parasitologically. As shown in Figure 2A, mice developed inflammatory lesions that progressed to small nodules and subsequently to ulcers with an appearance that was strikingly similar to the typical ulcerative lesions observed in human CL by *L(V)p*, consisting of a well-circumscribed ulcer, with a granular and crusted base, and indurated borders (Weigle and Saravia, 1996; Aronson et al., 2020). The incubation period after a 10^5^ promastigotes inoculum typically ranged from 2 to 3 weeks but can be extended up to 7 weeks when lower infective challenges are used (Figure 2B; Supplementary Figure S3). From week 3 to 6 postinfection, most of animals (usually over 90%) presented ulcers and lesions that looked similar in appearance and size among mice. By week 7, the average of lesion size stabilized, with some animals maintaining their lesion or even improving, and others exhibiting aggravation (Figure 2B), as indicated by the presence of larger ulcers with necrotic foci. Although overall few live parasites (0.1% of the inoculum) were isolated at day 1 and 7 postinfection, the vigorous parasite multiplication that took place in the following weeks preceding disease onset and progression demonstrate that survivors were sufficient to establish a productive infection (Figures 2A–D). Parasite growth stabilized at later timepoints (7 to 9 week postinfection), indicating effective control of replication by host (Figure 2D). Interestingly, in spite that the slope of parasite growth significantly flattened after 5 weeks postinfection, ulcerative lesions remained or aggravated in many of the animals (Figures 2B,D), indicating that the pathological processes that maintain ulcers and tissue damage persist even in the presence of an effective antiparasitic response during this chronic phase of the disease. Independent experiments in which extended follow-up was performed demonstrated the chronic, yet auto-resolutive, nature of this model (Supplementary Figure S4). While early (up to week 10 postinfection) lesion resolution is observed in very few animals, the majority exhibits large, often mutilating, chronic lesions that resolve slowly and require up to 24 weeks to heal completely (Figure 2B; Supplementary Figure S4).

Collectively, these results demonstrate that BALB/c mice infected id in the ear with UA-946 *L(V)p* develop an evident cutaneous disease that closely replicates human CL lesions. This adapted strain has been maintained and used in our laboratory for years with a remarkable reproducibility (Supplementary Figure S5). These two reasons prompted us to further characterize other aspects of the host response at this anatomical location.

### Lymphadenopathy and Parasite Dissemination in the Absence of Systemic Disease

**Figure 3:**
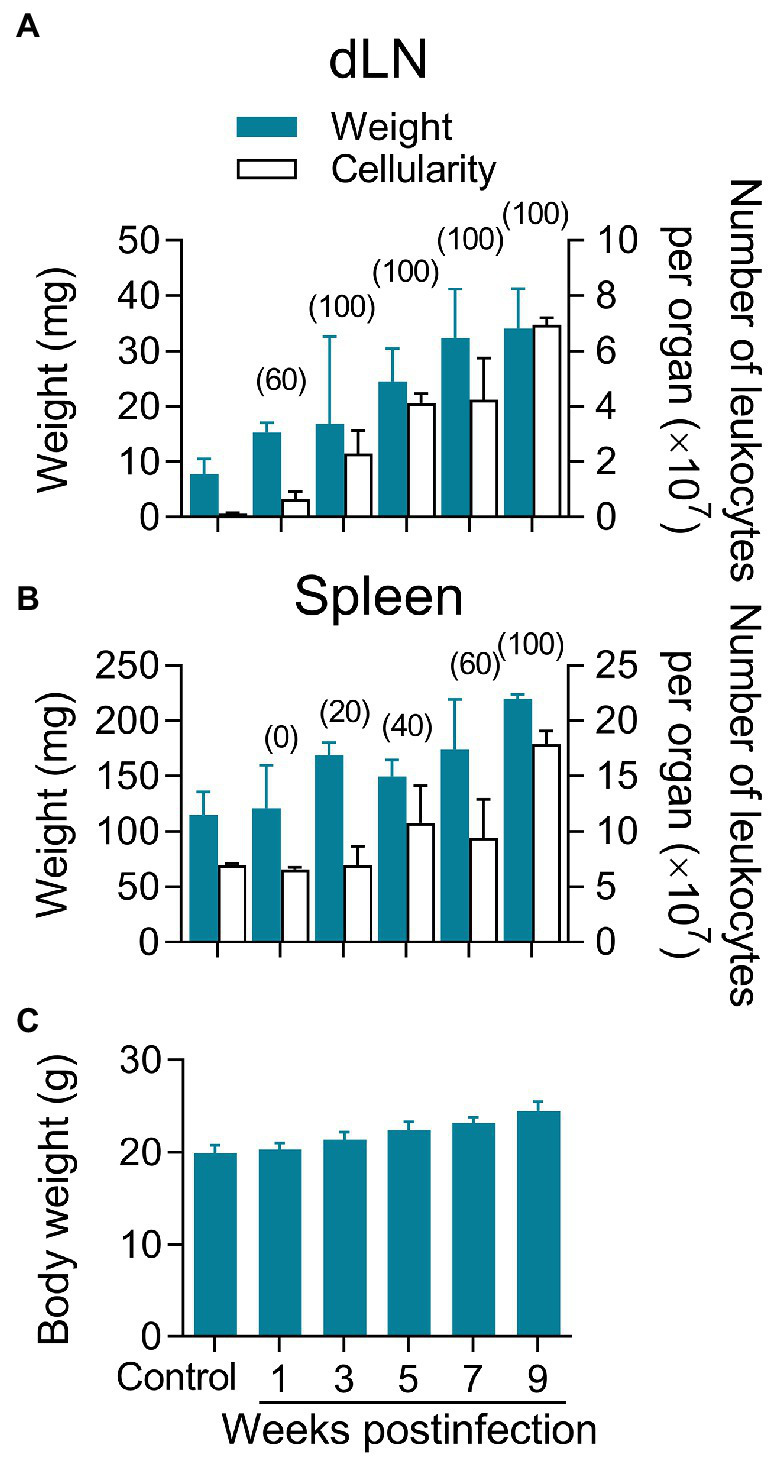
Lymphadenopathy and parasite dissemination in the absence of systemic disease. Mice infected as indicated in Figure 2 were used to remove the dLN in order to determine the weight and cellularity of the organ **(A)**. The spleens were also obtained for a similar analysis **(B)**. Blue bars indicate the weights, and white bars show the number of leukocytes **(A,B)**. Cell suspensions from both organs were also cultured to determine the percentage of mice harboring viable parasites at the indicated timepoint, which is shown in the top of the bars in parenthesis **(A,B)**. As an indicator of overall health, the total body weight per mouse was also determined at the indicated time **(C)**. A group of non-infected mice was also analyzed as controls for comparison purposes. Healthy BALB/c mice of the same age gained approximately 0.5 g of body weight per week, which lies within the normal ranges reported by the commercial supplier. Data correspond to individual analysis of 3–6 mice per time. The results are expressed as median ± interquartile range.

Regional lymphadenopathy is a common early clinical feature of CL by *L(V)* that can precede lesion appearance (Palma et al., 1991; Weigle and Saravia, 1996; Bomfim et al., 2007; World Health Organization, 2010). Compared to non-infected, *L(V)p*-infected mice had dLNs that progressively increased in weight along infection, which parallels an increase in the number of leukocytes (Figure 3A), suggesting progressive cell recruitment and/or proliferation in this organ. Maximal growth of dLN was observed at later timepoints of infection, which coincides with higher disease severity and parasitic loads (Figures 2, 3A). Mice infected with *L(V)p* in the footpad (Figures 1E,F) or the base of the tail (not shown) presented a similar kinetics of dLN swelling. Notably, viable parasites could be isolated from the dLN of most of mice as soon as the week 1 postinfection, which has been also documented in human cases as a demonstration of the early *L(V)* spread *via* lymphatics during primary infections (Weigle and Saravia, 1996). All animals were positive for parasite growth by week 3 postinfection, and this status persisted for the rest of the study (Figure 3A). A preliminary characterization of the dLN cells by flow cytometry demonstrated a rapid, progressive, and sustained recruitment of CD11c + CD11b- Gr1- and to a lesser extent of CD11c + CD11b + Gr1- myeloid cells, likely corresponding to dendritic cell subsets migrating from the periphery (Supplementary Figures S6A, S7A). This kinetics remarkably coincides with a tenfold increase in the absolute numbers of CD3+ CD4+, CD3+ CD8+, and CD3+ double-negative T cells observed during the study period (Supplementary Figures S6B, S7B) that most likely were the result of massive T cell expansion upon *L(V)p* Ag presentation. Although less accentuated, the weight and cellularity of the spleen also increased after infection, and maximal values were observed at the latest timepoint evaluated (Figure 3B). Furthermore, parasites also reached the spleen of some mice by the week 3 postinfection and were detectable in all mice at 9 weeks postinfection (Figure 3B), suggesting hematogenous besides lymphatic dissemination. Notably, the evident parasite dissemination to proximal and distal lymphoid organs was not associated with signs of systemic disease, since animals looked healthy and total body weight gain was normal and comparable to non-infected mice (Figure 3C). Mice infected at the footpad, or the base of the tail also remained free of clinical signs of systemic disease. Altogether, these findings demonstrate that cutaneous lesions in *L(V)p*-infected BALB/c mice are accompanied by regional lymphadenopathy and parasite dissemination but not by systemic clinical involvement, which again was reminiscent of the features of leishmaniasis by *L(V)* in immunocompetent humans as a systemic but non-life-threatening infection with a localized cutaneous presentation (Weigle and Saravia, 1996; Scorza et al., 2017; Burza et al., 2018).

### Antigen-Specific Adaptive Immune Response to *L(V)p* Infection

**Figure 4:**
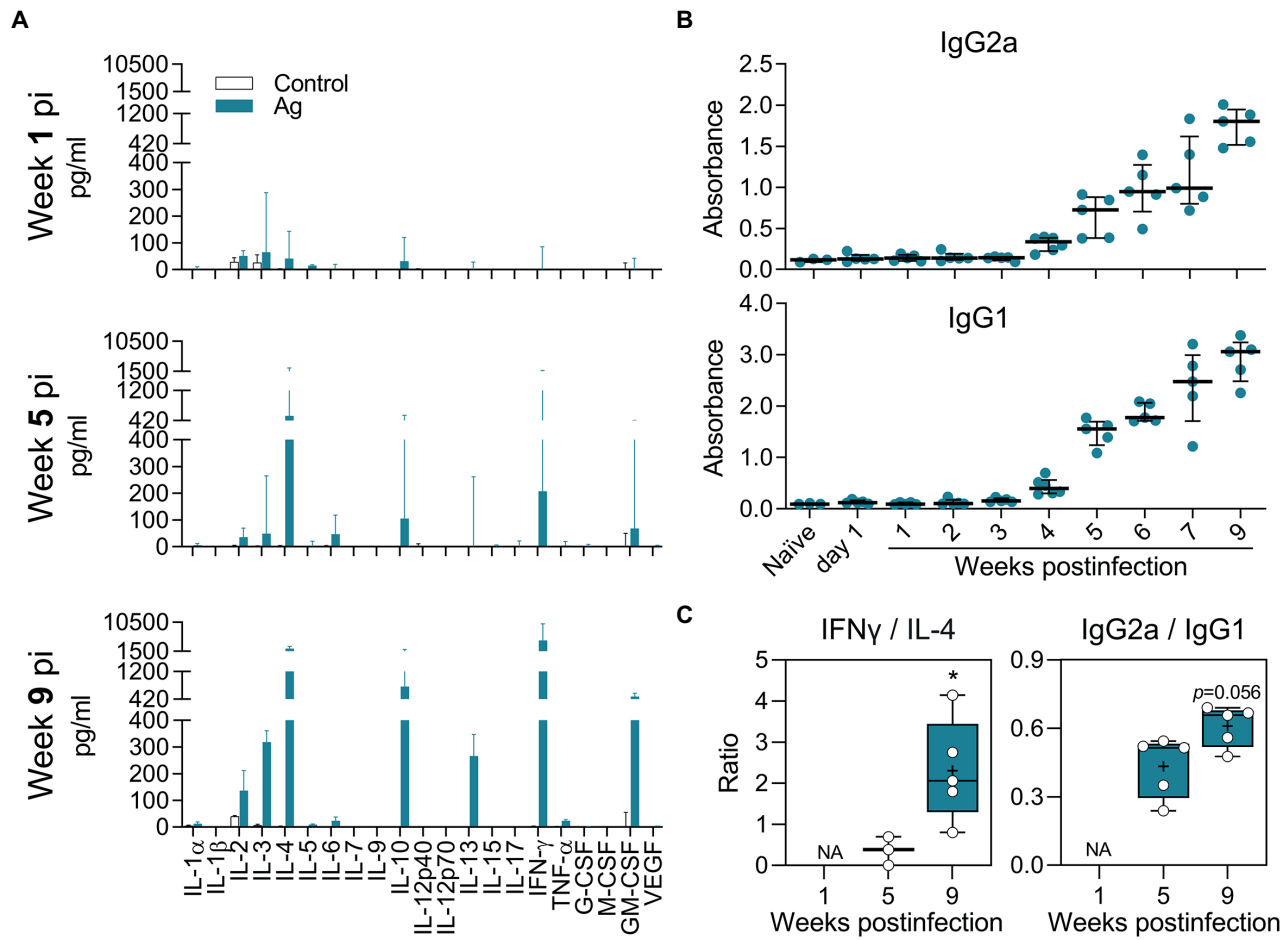
Antigen-specific cytokine and antibody response of *L(V)p*-infected BALB/c mice. Draining LNs from *L(V)p*-infected mice were obtained at the indicated timepoints to prepare cell suspensions. Cells were cultured in the absence (Control) or presence (Ag) of *L(V)p* total lysate. The concentration of the indicated cytokine secreted by dLN cells was determined by a multi-analyte Luminex platform and graphed as the median ± interquartile range **(A)**. Serum samples were also used to quantify the antigen-specific IgG1 and IgG2a response by ELISA **(B)**. Ab levels in individual mice are shown and the bars are the median ± interquartile range. IFNγ/IL-4 and IgG2a/IgG1 ratios were calculated and graphed as “box and whiskers” plots **(C)**. IFNγ/IL-13 and IFNγ/(IL-4 + IL-13) ratios exhibited a similar pattern to that presented in **(C)**. NA: not applicable. pi: postinfection. *n* = 3–6 mice/group. ^*^p < 0.05 (Mann–Whitney *U*-test in **(C)** comparing week 5 vs. 9).

The presence of parasites and the accumulation of immune cells in the dLN and spleen of *L(V)p*-infected mice indicated that active Ag presentation and lymphocyte activation/proliferation were taking place in secondary lymphoid organs after infection. Moreover, T cells compatible with Th1 (IFNγ-producing) and Th2 (IL-4-producing) were already present in the dLN as early as week 3 postinfection and its abundancy increased to peak at week 5–7 postinfection (Supplementary Figure S7C). It was therefore important to characterize the cytokine response upon Ag re-stimulation *in vitro*, as a surrogate of parasite-specific T cell response *in vivo*. By using a multi-analyte Luminex system, we screened for the secretion of cytokines and growth factors (Supplementary Table S3). As shown in Figures 4A, a mixed immune response was observed in the dLN. Although the levels of cytokine secretion at early times of infection (week 1 postinfection) were low or no detectable, the pattern remained with a more robust response at later timepoints. Type 1 (IL-2, IFNγ), type 2 (IL-4 and IL-13), and regulatory (IL-10) cytokine responses were observed upon Ag re-stimulation during the whole observation period. Interestingly, other cytokines, such as IL-3 and GM-CSF, were also detected in appreciable amounts. Splenocyte Ag stimulation induced a cytokine profile with a remarkable similarity to that observed in dLN. A notable exception was the strong induction of IL-6 in splenocytes compared to the very weak response observed in the dLN (Supplementary Figure S8; Supplementary Table S3). Although some differences in the kinetics and relative abundance of certain cytokines (such as IL-4, IL-13, and IL-5) were observed, a mixed Th1/Th2/regulatory/inflammatory response (IL-2, IFNγ, IL-4, IL-13, IL-10, and GM-CSF) was also present in mice infected in the footpad (Supplementary Figures S9A,B). The *L(V)p*-specific antibody response was also analyzed in animals infected in the ear or the footpad. We observed an early IgG1 response that increased along with disease progression until stabilization (or even decline at the late chronic stage) when the parasite multiplication is controlled (Figure 4B; Supplementary Figure S9C). In contrast, the Th1-related IgG2a response was delayed but steadily increased until the end the experiment.

In summary, *L(V)p*-infection elicited a mixed adaptive immune response in BALB/c mice, involving T cells that produce Th1, Th2, Treg, and inflammatory lymphokines, which replicates the combined Th1/Th2/Treg cytokine pattern observed in stimulated PBMCs from *L(V)p*-infected humans (Figure 4; Supplementary Figure S8; Bosque et al., 2000; Trujillo et al., 2002; Castilho et al., 2010; Díaz et al., 2010). Moreover, the IFNγ/IL-4 and IgG2a/IgG1 ratios calculated at late weeks postinfection compared to those calculated at earlier timepoints suggested that the early non-protective Ag-specific Th2 response is shifted toward a Th1-dominated response that enables parasite control (Figure 4C; Supplementary Figure S9D).

### *In situ* Changes Related to *L(V)p* Infection and Disease Development

**Figure 5:**
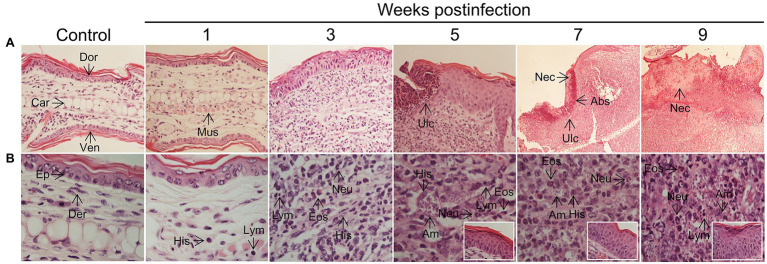
Histopathology of *L(V)p*-infected BALB/c mice ears. Representative photographs of hematoxylin/eosin-stained ear sections from the infected mice at the indicated week postinfection are shown. A photograph of a section from a normal non-infected mouse was also included (Control) for comparison. Magnifications are 40X for control and 1–5 weeks postinfection, and 10X for 7 and 9 weeks postinfection in the upper panel **(A)**. In the lower panel, a 100X magnification was used for all photographs **(B)**, to document the appearance of the dermis. Insets in the lower panel show the appearance of the epidermis. Note that no major changes were observed in the ears at the 1st week postinfection in the dermal compartment (no parasites, and very few histiocytes, granulocytes or lymphocytes could be identified), and no alterations were detected in the epidermis, which is consistent with the clinical, parasitological, and immunological findings at this time (Figures 2–4). At week 3 postinfection, however, an evident inflammatory infiltrate was observed in the dermis predominantly represented by granulocytes (primarily neutrophils but also eosinophils), amastigote-containing histiocytes and lymphoplasmocytes. Hypertrophic/hyperplasic muscle fibers could be observed in the dermis, and irregular acanthosis in the epidermis was also present in the areas where the inflammatory reaction was taking place. More pronounced changes that indicated aggravation of the inflammatory process were observed at subsequent times (5th–9th weeks postinfection). Heavily parasitized histiocytes were observed starting at the 5th week postinfection until the end of the experiment. A profuse lymphoplasmacytic and granulocytic infiltrate was also evident in the dermis, with granulocytes forming microabscesses, which were also present in the epidermis, presumably provoking ulcerations. Marked and progressive acanthosis and hyperkeratosis, the presence of necrotic areas in the dermis and epidermis and accentuated forms of all previously described changes, were common anatomopathological characteristics of the chronic phase (7–9 weeks) of the disease. All histopathological changes were restricted to affected skin areas since the zones of the ear where neither parasite replication occurred, nor leukocyte infiltrated was present, looked histopathologically normal (with only some evidence of muscle hypertrophy). Eos, polymorphonuclear eosinophil; Neu, polymorphonuclear neutrophil; Ep, epidermis; Der, dermis; Mus, muscle, Abs, abscess; Ulc, ulcer; His, histiocyte; Am, amastigote; Dor, dorsal face; Ven, ventral face; Car, cartilage; Nec, necrosis; Lym, lymphoplasmocyte.

Studies with human biopsy specimens taken from *L(V)p*-infected patients reported epidermal hyperplasia/hyperkeratosis; diffuse dermal infiltrate of lymphocytes, histiocytes, neutrophils, eosinophils and plasmocytes; and the presence of amastigotes, necrotic foci, granulomas and resolution-related epithelioid and giant cells as usual anatomopathological characteristics (Palma et al., 1991; Weigle and Saravia, 1996; Palma and Saravia, 1997; González et al., 2018). Immunohistological phenotyping further confirmed the cellular identity of the major infiltrators and additionally discriminated the lymphocyte compartment into B and T cells, CD4+ and CD8+ T cells, and Th1, Th2, Th17, and Treg cell subpopulations as potential drivers of pathology and/or immunity (Isaza et al., 1996; Palma and Saravia, 1997; Gonzalez et al., 2020a,b). Although the magnitude and pattern of infiltrates might be heterogeneous in human samples, our histopathological and flow cytometric analysis in the ears of *L(V)p*-infected mice evidenced most of those characteristics (Figure 5; Supplementary Figure S10). Ear sections evidenced a progressive recruitment of inflammatory cells (Figure 5) that paralleled parasite multiplication and clinical lesion appearance and aggravation (Figure 2). Neutrophils, eosinophils, parasitized histiocytes, lymphocytes/lymphoplasmocytes, as well as acanthosis, were consistently observed and its abundance accentuated as disease progressed. Most severe pathological changes, such as massive leukocyte infiltration, abundant presence of heavily parasitized macrophages, epidermal hyperplasia/hyperkeratosis, and the appearance of necrotic areas, were observed at 7–9 weeks postinfection (Figure 5), timepoints at which the adaptive response was already vigorous (Figure 4) and right after the temporal window in which robust parasite multiplication took place (Figure 2). These histopathological findings were in line with our preliminary flow cytometry analysis from ear macerates, which revealed the massive and progressive infiltration of myeloid cells phenotypically compatible with granulocytes, and mononuclear phagocytes (Supplementary Figure S10A). Flow cytometry also allowed to evidence the emigration of myeloid cells phenotypically compatible with skin resident dendritic cells, which seem to be the cells appearing at the same times in the dLN (Supplementary Figure S7), presumably transporting parasites and parasite antigens (see section “Lymphadenopathy and Parasite Dissemination in the Absence of Systemic Disease” above). CD4+ and CD8+ T cells also infiltrate the infected ears with maximal amounts observed at 5 weeks postinfection (Supplementary Figure S10B). Both IFNγ- and IL-4-producing CD4+ T cells, as well as IFNγ-producing CD8+ T cells, were detected in infected but not in control mice (Supplementary Figure S10C), further suggesting that a mixed Th1/Th2 response also characterizes the *in situ* response to *L(V)p* in BALB/c mice. Histopathological analysis of footpad sections at week 7 postinfection showed that *L(L)m* induces massive inflammation and tissue destruction, whereas *L(V)p* did not alter the global tissue architecture and induces only moderated and limited changes (see Supplementary Figure S11 and legend for details).

Collectively, results presented thus far demonstrate that the development of a progressive inflammatory response and the establishment of a robust mixed Ag-specific adaptive immune response are two key events associated with *L(V)p* infection and pathogenesis in BALB/c mice.

### Exploiting the Model to Evaluate Pharmacological and Immunological Interventions

**Figure 6:**
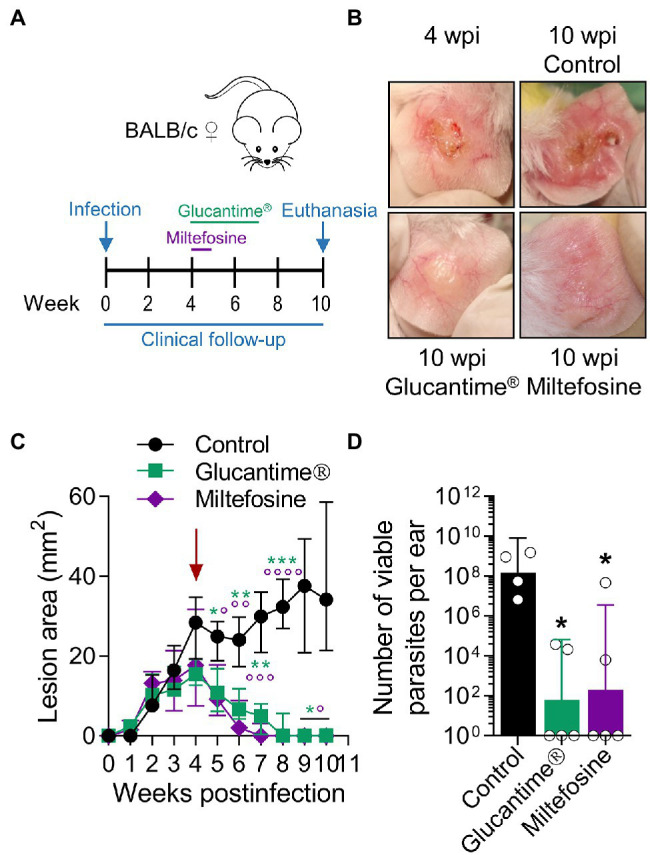
Using the *L(V)p*-BALB/c model to investigate pharmacological interventions. BALB/c mice were infected id in the ear with 10^5^ stationary promastigotes and lesion development monitored weekly **(A)**. Once lesions were established at the week 4 postinfection (**B**, *left upper corner*; **C**, *arrow*), mice were treated ip with Glucantime^®^ (500 mg/kg, once a week, 4 weeks) or orally with Miltefosine (20 mg/kg/day, five consecutive days) as indicated **(A)**. Representative photographs taken at the indicated timepoints are shown **(B)**. The therapeutical effect of the agents was clinically monitored measuring the size of the lesions during the subsequent weeks **(C)**; asterisks and small circles correspond to *p*-values from the statistical test comparing control versus Glucantime^®^ and Miltefosine, respectively. The burden of viable parasites in the ears at the end of the experiment (week 10 postinfection) was analyzed by a limiting dilution assay **(D)**. Data in **(C,D)** are presented as the median ± interquartile range (*n* = 5–12 mice) and the geometric mean ± 95%IC (*n* = 4–5 mice), respectively. wpi: weeks postinfection. ^*^p < 0.05, ^**^p < 0.01, ^***^p < 0.001, ^****^p < 0.0001 (Mixed-effects analysis with Geisser–Greenhouse correction and Bonferroni’s multiple comparisons *post-hoc* test for multiple comparison through time; Kruskal–Wallis test with Dunn’s multiple comparison *post-hoc* test). A repetition chemotherapy experiment with Glucantime^®^ in which an extended clinical follow-up was performed, indicated that remaining mild erythema/swelling observed in some animals at week 10 postinfection completely resolved within the following 2–3 weeks and only a rough/bright clear appearance, most likely representing scarification, was observed. Results are representative of three independent experiments performed with Glucantime^®^, whereas the experiment with Miltefosine was performed only once.

Since antimonials and Miltefosine are first-line drugs used for leishmaniasis (Reithinger et al., 2007; Aronson et al., 2020), we tested the clinical and parasitological response to these agents in BALB/c mice with established *L(V)p*-induced LCL. Mice treated with antimonials were cured (as defined by the complete disappearance of the ulcer and the re-epithelialization of the affected skin) within 3–7 weeks and had about a 10^5^–10^6^-fold reduction in the parasitic load when infected in the ears (Figures 6A–D), as well as in the footpad (Supplementary Figure S12A) or the base of the tail (Supplementary Figure S12B). Miltefosine was also leishmanicidal *in vivo* and induced cure of *L(V)p*-infected mice (Figures 6A–D). This indicates that common drugs used to treat *L(V)p* infection in humans also promote disease resolution in our animal model.

**Figure 7:**
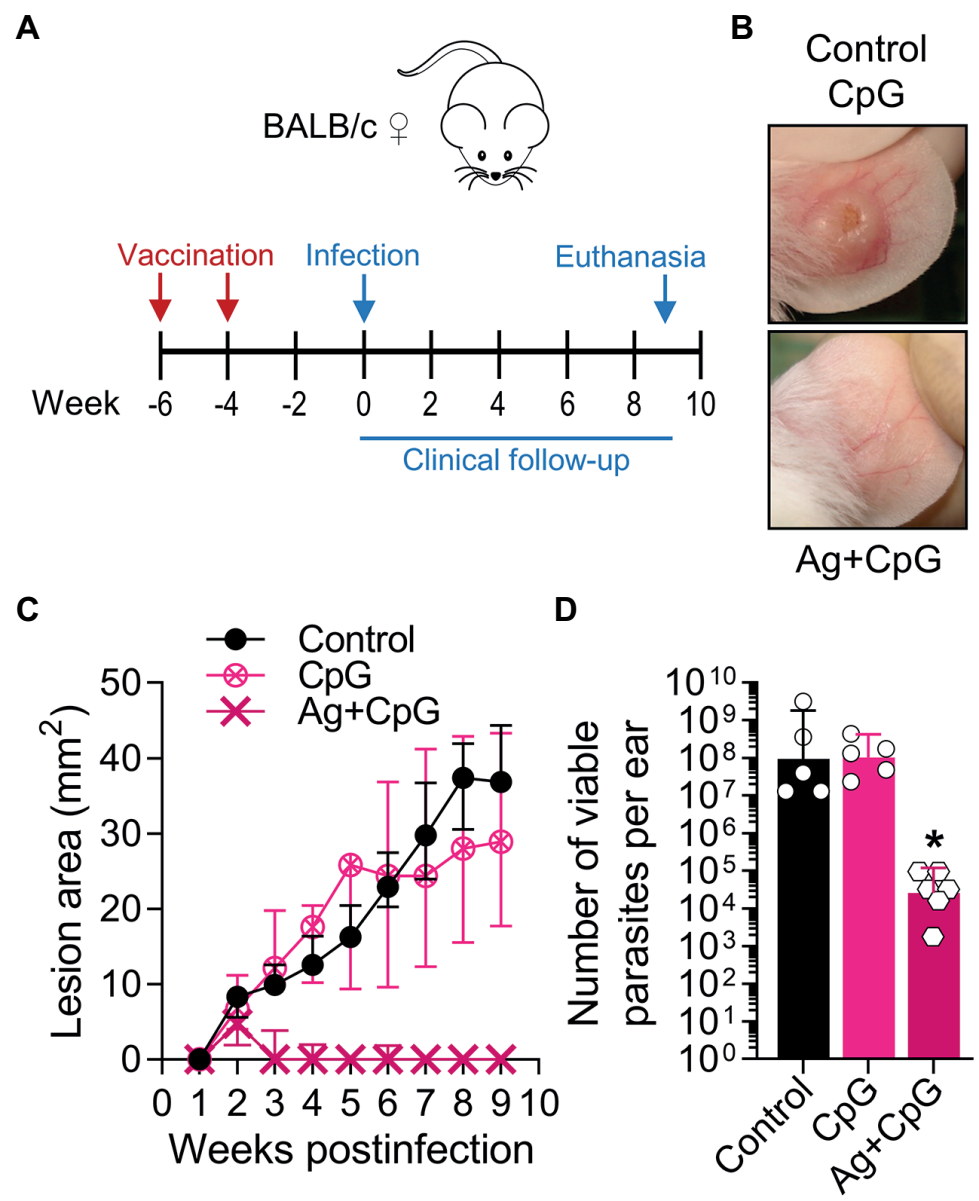
Using the *L(V)p*-BALB/c model to investigate immunological interventions. BALB/c mice were vaccinated with Ag + CpG (12.5 μg/6.25 μg sc in the base of the tail) and boosted with the same dose 2 weeks later, to be infected in the ear dermis with 10^5^ *L(V)p* stationary promastigotes 4 weeks after the boost **(A)**. Mice were followed weekly to monitor lesion growth. Mice injected with CpG (6.25 μg) or PBS were used as controls. Representative photographs of the ears from Ag + CpG-vaccinated mice and controls at the week 6 postinfection are shown **(B)**. The kinetics of lesion progression after infection is graphed **(C)** as the median ± interquartile range. From week 2 postinfection, there was a statistically significant difference between control and Ag + CpG. The number of viable parasites at the site of infection was determined at the end of the experiment (week 9 postinfection) by a limiting dilution assay and graphed **(D)** as the geometric mean ± 95%CI. *n* = 5–6 mice. A similar efficacy of Ag + CpG has been observed in repetition experiments, with more than 90% of mice protected from clinical disease and a 10^3^–10^6^-fold reduction in parasitic loads. wpi: weeks postinfection. ^*^p < 0.05 (Two-way ANOVA with Geisser–Greenhouse correction and Bonferroni’s multiple comparisons *post-hoc* test for multiple comparison through time; Kruskal–Wallis test with Dunn’s multiple comparison *post-hoc* test).

We also tested CpG-containing oligodeoxynucleotides, a synthetic immunomodulatory agent that has been shown to deviate the immune response toward a protective Th1-type cytokine pattern and promote parasite control when used at low dose (Zimmermann et al., 1998, 2008; Raman et al., 2012). We found that the dermal co-delivery of CpG with *L(V)p* infective promastigotes prevented the development of cutaneous lesions in BALB/c mice and served also as a protective adjuvant when combined with total or membrane Ag (Supplementary Figure S13). Interestingly, CpG alone was sufficient to significantly protect BALB/c mice from an infective challenge when administered 2 weeks before infection, suggesting that CpG pre-treatment triggered a protective effect in the absence of leishmanial Ag lasting for at least 2 weeks (Supplementary Figure S13). Therefore, we performed vaccination experiments in which the immunizing Ag + CpG formulation was administered following a homologous prime-boost scheme, and the infective challenge administered 4 weeks after the boost (Figure 7A). A strong clinical and parasitological protection was observed that required the presence of both the adjuvant and the antigen (Figures 7B–D), indicating the potential of a TLR9-mediated pathway of protective adjuvanticity for vaccine development against this species. Collectively, these results confirm the utility of this model for the preclinical assessment of potential chemo/immunotherapeutic and prophylactic agents against *L(V)p* CL.

### A 28–30 kDa Protein Fraction of the *L(V)p* Proteome Protects From an Infective Challenge

Having demonstrated CpG as a protective adjuvant and the utility of our model in a vaccination setting, we initiated efforts to identify defined Ag as vaccine candidates using preparative proteomics. Total *L(V)p* Ag was fractioned by electroelution (Supplementary Figure S14A), and although sufficient protein yield for *in vivo* testing was obtained only for few fractions, results indicated that a fraction containing proteins in the 29–30 kDa range reproduced (Supplementary Figures S14B,C) the protective effect of the total lysate (Figure 7). We then shifted to a manual fractionation method to obtain fractions in amounts sufficient to perform a complete *L(V)p* proteome screening. From the 16 fractions obtained (Supplementary Figure S15A), 15 were tested *in vivo* using our mouse model, and although several fractions induced a significant clinical protection, the F9 (which contains proteins in the 28–30 kDa range) was again the most protective (Supplementary Figure S15B).

**Figure 8:**
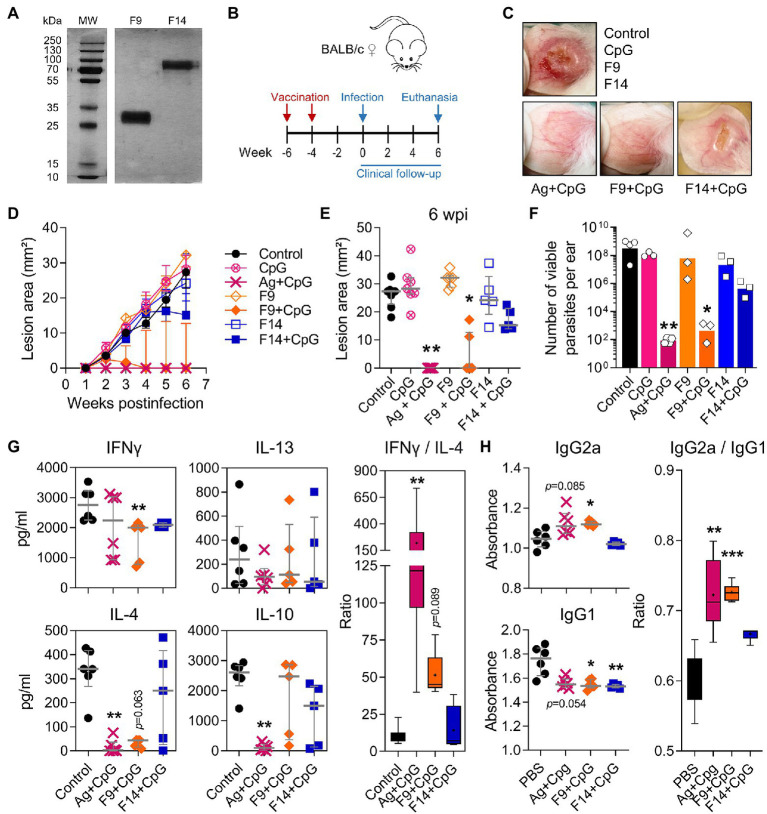
A 28–30 kDa protein fraction of the *L(V)p* proteome that protects against an infective challenge. In an independent experiment but following the same strategy presented in Supplementary Figure S15, the fractions F9 and F14 from the *L(V)p* promastigote proteome were obtained and run in SDS/PAGE gels. The photograph of a silver-stained gel presented in **(A)** confirmed that yield, purity, and integrity of the fractions were appropriated for *in vivo* testing. Mice were then vaccinated sc on the back with 6 μg of the protein fraction in combination with 3.5 μg CpG and boosted 2 weeks later with the same preparation to be infected with *L(V)p* 4 weeks after the boost **(B)**. PBS- or Ag + CpG-injected mice were used as negative and positive controls, respectively. Representative photographs illustrating the appearance of the infected ears in the different experimental groups at the 6th week postinfection are shown **(C)**. Lesion size was registered weekly and graphed as the kinetics of lesion growth **(D)** or the size of the lesion in individual mice at the 6th week postinfection **(E)**. Animals were sacrificed at the 6th week postinfection, and the ears used to quantify the numbers of viable parasites by limiting dilution assay **(F)**. Cell suspensions from dLN were stimulated with *L(V)p* Ag and the amounts of the indicated cytokine determined by ELISA **(G)**. The IFNγ/IL-4 ratios were calculated and graphed (**G**, *right*). Serum samples were also obtained and used to quantify the circulating levels of *L(V)p*-specific IgG1 and IgG2a antibodies **(H)**. IgG2a/IgG1 ratios were also calculated and graphed (**H**, *right*). Data are shown as the median ± interquartile range (**D,E,G,H**; *n* = 4–6 mice per group), or the geometric mean ± 95%CI (**F**; *n* = 3 pools from 5 to 6 mice per group). Ratios presented in **(G,H)** are graphed as “box and whiskers” plots. MW: molecular weight. wpi: weeks postinfection. ^*^p < 0.05, ^**^p < 0.01, and ^***^p < 0.001 (Kruskal–Wallis test with Dunn’s multiple comparison *post-hoc* test).

In a third experiment, we compared the protective F9 with the less-protective F14 fraction (Supplementary Figure S15; Figure 8A) and used PBS and total Ag as negative and positive controls, respectively. Two vaccine injections separated 2 weeks apart with F9 + CpG (Figure 8B) were sufficient to protect mice clinically and parasitologically from a *L(V)p* challenge 4 weeks after the boost (Figures 8B–F), and protection required the presence of both parasite antigens (F9) and CpG adjuvant (see groups CpG alone and F9 alone in Figures 8C–F). While the level of protection induced by F9 + CpG was comparable to that induced by total Ag + CpG, the F14 + CpG vaccine, as anticipated, did not confer significant protection. F9 + CpG-induced protection was related to a particular *L(V)p*-specific cytokine and antibody profile (Figures 8G,H). Counterintuitively, IFNγ production seemed to be lower in vaccinated groups (Figure 8G); however, when normalized to the lesion size (as surrogate of antigenic load that strongly correlates with viable parasite burden; Supplementary Figure S16A), it emerged that vaccination with Ag + CpG induced significant amounts of this key antimicrobial cytokine (Supplementary Figure S16B). It was interesting that Ag + CpG also induced IL-13 but no IL-4 and that the pattern of F9 + CpG-vaccinated mice resembled that from Ag + CpG-vaccinated animals, whereas the pattern induced by F14 + CpG resembled more that observed in control non-vaccinated mice (Supplementary Figure S16B). In a similar manner, IFNγ/IL-4 and IgG2a/IgG1 ratios also indicated that vaccination with F9 + CpG, but not with F14 + CpG, promoted a significant shift toward a Th1-type of response which characterizes protected animals vaccinated with Ag + CpG (Figures 8G,H, *right panel*). Finally, in a separated set of experiments, we used the model to confirm that mice vaccinated with F9 are protected not only from a primary, but also from a secondary infection (Supplementary Figure S17), and that fine dosing of F9/adjuvant is critical for optimal induction of protection (Supplementary Figure S18). Collectively, in these experiments we exploited the *L(V)p*-BALB/c model to identify a fraction of the *L(V)p* proteome encompassing potential antigenic candidates for the preclinical development of a molecularly defined vaccine and to explore immunological surrogates of protection.

## Discussion

Although mouse models of human leishmaniasis caused by most *Leishmania* species have been implemented in many laboratories worldwide permitting significant advance of science in the field, attempts to develop a murine model of *L(V)p* infection that reproduces the manifestations of the human disease have been relatively unfruitful. In this article, we presented the first ulcerative mouse model of *L(V)p* infection, the characterization of the disease at the stages of parasite multiplication and control, and examples of its use for preclinical pharmacological testing and vaccine development. The availability of this new model warrants further research that could expand the understanding of the immunobiology of *Leishmania* infections and promote translational work for a better control.

In pioneering efforts seeking to establish a murine model of *L(V)p* infection, researchers injected extremely high amounts of parasites (10^7^–10^8^ parasites) and reported inflammatory lesion formation (Neal and Hale, 1983; Guevara-Mendoza et al., 1997). Subsequent attempts, however, were discouraging, since no reproducible and predictable lesion formation could be induced (Hommel et al., 1995; Travi et al., 2002; McMahon-Pratt and Alexander, 2004; and our laboratory observations). This led to the perception that mice were not reliable to model infections with *L(V)p* and with *L(V)* organisms more generally. More recently, efforts in several laboratories indicated that under particular experimental conditions mice can develop, yet small and self-limited, measurable and reproducible skin lesions after infection with *L(V)* isolates (de Moura et al., 2005; Sousa-Franco et al., 2006; Castilho et al., 2010; de Oliveira and Brodskyn, 2012; Novais et al., 2013; Pena DaMata et al., 2015; Borges et al., 2018; Hartley et al., 2018; de Carvalho et al., 2019). The selection-adaptation strategy implemented in our laboratory allowed the establishment of a suitable *in vivo* system that recapitulates the essential human clinicopathological and immunoinflammatory responses to *L(V)p* as a member of the *L(V)* subgenus and that follows three phases: (1) an acute phase (from day 0 to week 5–6) in which a short early silent period of parasite establishment takes place, followed by a period of robust parasite multiplication, lesion appearance, and progression to ulcer. (2) An intermediate chronic phase, lasting from week 5–6 to week 11–12, in which massive tissue damage is observed often leading to partial or total mutilation. (3) A final stage of lesion resolution, lasting up to 22–24 weeks, in which lesion is healed and inflammation resolved. While most of available murine models of *L(V)* infections in BALB/c and C57BL/6 mice exhibit an inflammatory acute phase with variable magnitude and duration followed by rapid resolution/healing, our model is unique in presenting the intermediate phase, making it particularly useful to investigate the chronic disease and immunopathology. Moreover, C57BL/6 mice were resistant to *L(V)p* as indicated by the very small lesions accompanied by low parasitic loads observed after infection in the ear (not shown). This clear cut of mouse susceptibility/resistance to UA-946 *L(V)p* resembles the classical model of *L(L)m* but without the disadvantage of the systemic, visceralizing, and lethal disease observed in BALB/c mice, and thus, *L(V)p* appears to better model not only *L(V)* infections particularly, but CL in general.

Importantly, progression to the mutilating chronic phase in our model occurs in the absence of further parasite multiplication because parasite loads after week 10 postinfection were always below the maximal 10^9^ and usually in the 10^4^–10^8^ range (not shown), a consistent observation in many independent experiments performed in our laboratory during years, indicating that persistence/progression of disease is the result of an immunopathological process. That ulceration was the result of lymphocyte-driven immunopathology was indicated by the slow-progressing non-ulcerative lesions observed in SCID BALB/c mice infected with a similar UA-946 *L(V)p* inoculum (not shown). This is of importance since a hallmark of *L(V)* infections in humans is the propensity to induce chronic tissue-damaging immunopathology in the context of effective parasite control. While the immunopathological mechanisms operating in *L(V)* infections have been enigmatic for many years, two relevant pathways have been recently described. In one, cytotoxic activities involving CD8+ T and NK cells linked to inflammatory executors such as NLRP3 inflammasome, IL-1β, and neutrophils mediate exacerbated tissue damage in response to *L(V)b* infection (Novais et al., 2021; Carvalho et al., 2022). In the other, the innate immune recognition of the viral RNA present in *L(V)g* isolates harboring the *Leishmania* virus LRV1 (or *via* exogenous viral coinfections) triggers a type I IFN response, immunopathology, chronicity, and metastasis (Rossi and Fasel, 2018b). Whether these two mechanisms also operate during pathogenic response to *L(V)p* is an open question to be addressed in the future. The *L(V)p* model presented here, in which no special manipulation of the host (such as the CD8+ T cell reconstitution of immunodeficient mice or the use of IFNγ-deficient mice) or additional innate inflammatory trigger in the infective inoculum (such as the viral endosymbiont) are required to exhibit the chronic and tissue damaging phenotype, could be convenient to further investigate LRV1/cytotoxicity-dependent or LRV1/cytotoxicity-independent mechanisms of pathogenesis and elucidate the relative contributions of microbial- and host-derived factors to the intriguing and distinct pathophysiology of *L(V)* infections. Moreover, given the known link between disease chronicity and refractoriness to treatment, the preclinical testing of host- and parasite-directed strategies aimed to reduce deleterious inflammation is necessary, and this model appears suited for this purpose.

We observed that infection in three different anatomic locations leads to effective establishment and multiplication of *L(V)p*, although id/ear delivery was optimal both at mimicking the human disease and at exhibiting consistency and reproducibility. While the influence of the route/site of *Leishmania* infection on the clinical outcomes has been long documented (Kirkpatrick et al., 1987; Loeuillet et al., 2016), the underlying mechanisms are incompletely understood. Ribeiro-Gomes et al. showed that id but not sc delivery of *L(L)m* leads to the rapid recruitment of high amounts of neutrophils that efficiently capture parasites, providing appropriate host cells that assure successful parasite establishment and subsequent multiplication (Ribeiro-Gomes et al., 2014). The study additionally indicated that it is the route (id) rather than the anatomical site (ear or footpad), what dictates the higher neutrophil response to dermal injury and that the effective dose achieved after id/ear injection could be reached *via* sc/footpad infection by increasing (approximately tenfold) the size of the inoculum. Our *L(V)p* promastigote inoculum was 10^5^ for id/ear injection and 10^6^ for sc/footpad injection, and yet tissue destructive versus mild non-ulcerative inflammation, respectively, were observed. This, together with the different outcomes after UA-946 *L(V)p* delivery using the same route (sc) but different site (base of the tail and footpad), which was also observed in hamsters infected with *L(V)p* id in the foot versus id in the snout (Osorio et al., 2003), and the similar outcome after using similar site (foot) but different routes (sc route in the present study and id route in the study by Castilho et al., 2010), indicate that site-related effects are major contributors to the different disease presentation. While our results indicated that the overall pattern of T cell response in the LN draining the ear and the footpad was similar, detailed analysis revealed some differences, such as the kinetics of the Th2 cytokines IL-4 and IL-13, with footpad infection exhibiting early transient high levels and ear infection inducing high levels that are sustained and even increasing at the chronic phase, which is consistent with the abundant literature pointing to the type of adaptive immune response induced (whether biased toward Th1, Th2, or regulatory) as a determining factor of the distinct site-related outcomes (Kirkpatrick et al., 1987; Nabors and Farrell, 1994; Nabors et al., 1995; Baldwin et al., 2003; Tabbara et al., 2005; Mahmoudzadeh-Niknam et al., 2013). A recent report showed that TLR7-dependent effector functions of neutrophils control early parasite replication and subsequent disease progression without apparent alteration in the CD4+ T cell response after id/ear (but not sc/footpad) *L(L)m* infection (Regli et al., 2020), reinforcing the initially unanticipated role of the innate response. How the interplay between innate and adaptive immune cells is modulated in different sites of the skin to promote diseases with different presentation, duration, and severity, and whether this is also a *Leishmania* species-specific phenomenon is also to be investigated in the future.

In the *L(V)p*-BALB/c model reported by Castilho et al., localized lesions are observed when animals are infected id in the dorsal side of the foot skin with late stationary (day 20, Percoll-purified) promastigote cultures containing amastigote antigen-expressing putative metacyclic forms (Castilho et al., 2010). As explained, our BALB/c mice infected sc in the footpad exhibited a disease that was similar in kinetics and appearance, which, together with the prolific parasite replication observed early after infection and during disease onset, and the persistence of relatively high parasite burdens in the chronic phase in both studies, suggests that skin in this anatomical location might be particularly refractory to inflammation-induced tissue damage and ulceration. By shifting the site of infection to the ear, we provoked progressive inflammatory lesions that resulted in tissue damage and ulcer formation. It will be interesting to determine whether the inoculum used by Castilho and collaborators is able to induce ulcerative lesions in other anatomical locations such as the ear, and, conversely, whether our UA-946 *L(V)p* inoculum (obtained *via* selection-adaptation) is also enriched in amastigote antigen-expressing infective forms. In pilot experiments, we performed Percoll-purification of live promastigotes from very late (day 15) stationary *L(V)p* cultures before infection of the ears, and no significant increase in lesion size or severity was found as compared to early-stationary or late-logarithmic cultures (not shown). This was consistent with the less strict growth phase dependency of infectivity exhibited by *L(V)p* promastigotes (Saravia et al., 1990) and indicates that no additional properties appear to be acquired by UA-946 *L(V)p* during long-term culture that further promotes its virulence *in vivo*, but rather, that adaptation to mouse tissue was sufficient to maintain its ability to reliably induce ulcerative chronic lesions in BALB/c mice. Further research is required to better understand whether and how metacyclogenesis influences *L(V)p* infectivity, pathogenicity, and healing. A second aspect of the model reported by Castilho et al. (2010) and subsequent mechanistic and interventional studies (Jayakumar et al., 2011; Ehrlich et al., 2014, 2017; Siefert et al., 2016), which applies also to our footpad model, is that disease is defined on the basis of the inflammation (increased thickness) of the foot linked to parasite multiplication but not to tissue damage (such as loss of tissue, presence of necrosis or microabscesses, etc.). Thus, conclusions derived from studies using the foot models are compelling regarding the antiparasitic immune response but limited at deciphering the events underlying immunopathology beyond its relationship to parasite growth/restriction promoted by the immune system. It would be interesting to use the ear UA-946 *L(V)p* model to confirm whether the parasite-promoting activities of IL-13, IL4R, and IL-10 and the host-protective parasite-restrictive actions of Treg cells revealed by the foot model (Castilho et al., 2010; Ehrlich et al., 2014) are also relevant during the immunopathological chronic phase.

Our Luminex multianalyte cytokine analysis not only confirmed that the antigen-specific cellular response elicited by *L(V)p* in BALB/c mice is comparable to that of humans, but also allowed us to identify GM-CSF (and to a lesser extent IL-3) as a cytokine vigorously secreted after restimulation. We consider this observation of particular interest for two reasons: Firstly, because among the growth factors tested, GM-CSF was selectively induced in our experiments. It is known that GM-CSF exerts non-redundant activities during inflammatory responses in tissues, and, in this context, its classical role in phagocyte survival, activation, and subsequent triggering of antimicrobial effector mechanisms has been the basis to test GM-CSF as an adjunct therapy in CL (Almeida et al., 1999; Santos et al., 2004; Machado et al., 2021). A more recently recognized role of GM-CSF in wound healing (Krzyszczyk et al., 2018) and the apparent participation of the skin wound healing response in cutaneous leishmaniasis (Baldwin et al., 2007) reinforce the potential of GM-CSF-based interventions for CL management. However, its precise role in leishmaniasis is largely unknown and currently contradictory, since GM-CSF neutralization in infected mice exacerbated the disease in one study (Murray et al., 1995) whereas mice rendered unresponsive to this factor exhibited enhanced resistance in another (Scott et al., 2000). This is not surprising, since this factor can be proinflammatory, tolerogenic, or even immunosuppressive depending on the inflammatory context (Zhan et al., 2019), indicating that a better understanding is required to fully harness its therapeutic potential. And secondly, because we observed that GM-CSF was produced in an antigen-dependent manner, in amounts comparable to other relevant Th1-, Th2-, and Treg-associated lymphokines and with a pattern mirroring that of IFNγ, which points toward a biologically relevant role of these putative GM-CSF-producing T cells in our model. GM-CSF-producing Th cells have been shown to mediate a tissue-damaging pathological role during aseptic chronic inflammation (Hartwig et al., 2018; Ingelfinger et al., 2021) and also appear to mediate resistance to *Mycobacterium tuberculosis* (Rothchild et al., 2017). Whether persistence or increased activity of these cells could also mediate immunopathology in chronic infections is currently unknown but appears an interesting possibility to be explored. Our observation that, like IFNγ, GM-CSF production was maximal when maximal ear ulcerations were present (week 9 postinfection) argues in favor of this possibility. Alternatively, potential tolerogenic and reparative actions of GM-CSF could promote inflammation resolution and healing. Future work could help to elucidate the sources and targets, and the spatiotemporal conditioning factors that influence beneficial versus deleterious actions of this intriguing growth factor, and to illuminate the development of more rational immunotherapeutic strategies.

We further validated pharmacologically the model with two major chemotherapeutic agents used to treat leishmaniasis. In contrast to other *L(V)* models (de Moura et al., 2005; Sousa-Franco et al., 2006), both the magnitude/duration of disease and the considerable parasitic burden observed in UA-946 *L(V)p*-infected ears rendered our model more suited to study potential antiparasitic and clinical effects. A significant *in vivo* leishmanicidal effect that induced subsequent resolution of lesions was evidenced both with Glucantime^®^, the agent used to successfully treat the CL patient from whom the UA-946 *L(V)p* was isolated, and with Miltefosine, which has been increasingly used to treat CL in the New World (Pinart et al., 2020). Likewise, since host-directed immunotherapeutic interventions have been used to treat leishmaniasis (Mota et al., 2021) and are gaining special attention (Novais et al., 2021), we also confirmed that low dose of the TLR9 agonist CpG protected mice clinically and parasitologically from *L(V)p* CL when co-delivered with the challenge or when used as an adjuvant of total *L(V)p* antigen in a vaccination setting (Zimmermann et al., 1998, 2008; Raman et al., 2012). Moreover, the model allowed us to identify a protective 28–30 kDa *L(V)p* subproteome (termed F9) *via* forward vaccinology and confirm that F9 induces a shift toward a Th1 response. Since IFNγ-mediated responses are a prerequisite for *Leishmania* vaccine efficacy, *L(V)p*-specific IgG2a/IgG1 Ab ratios could be used as a simple surrogate to identify IFNγ-inducing proteins in future screenings of F9 subfractions. These and other efforts are in course in our laboratory with the goal of identifying, formulating, and testing candidates for developing a molecularly defined vaccine.

## Conclusion

In conclusion, we developed a mouse model of human CL that meets three major requirements: (1) close similarity in the clinical manifestations (presence of ulcerative and lasting skin lesions, lymphadenopathy, no systemic involvement), (2) similarity in the host response (robust inflammatory response, robust mixed Th1/Th2/Treg-like antigen-specific response), and (3) similarity in the acknowledged etiophysiopathology (parasite infection, multiplication, dissemination, and persistence; robust immune response with parasite containment and tissue damage; ultimate healing). This practical model replicates most of the findings in human cases of CL by *L(V)* generally and *L(V)p* particularly, which can be of utility to improve our understanding of leishmaniasis pathogenesis. Moreover, the rapid parasite multiplication and onset of measurable disease within 3–4 weeks of infection and the validated response to drug and immune interventions enable the model for pharmacological testing. Future transgenic modifications of UA-946 *L(V)p* would facilitate real time *in vivo* imaging of parasite growth and dissemination, making this model a suitable tool for a robust preclinical screening of drugs (Caridha et al., 2017, 2019) or vaccines, with the purpose of accelerating the development of better interventions. Since *L(L)m*-BALB/c is a poor CL model that leads to systemic lethal disease, our *L(V)p*-BALB/c mice model could be advantageous both biologically and ethically to study dermotropic *Leishmania* parasites.

## Data Availability Statement

The original contributions presented in the study are included in the article/Supplementary Material, further inquiries can be directed to the corresponding author.

## Ethics Statement

The animal study was reviewed and approved by Comité Institucional para el Cuidado y Uso de Animales, Universidad de Antioquia.

## Author Contributions

JR-P conceived, designed, and directed the study. JR-P, NM-D, and AG designed and planned the experiments. NM-D, AG, and NG-V conducted the experiments. MR performed the histopathological analysis. MO conducted the *in vivo* screening of human *L(V)p* isolates. JR-P, NM-D, AG, NG-V, and DB-E analyzed the results and interpreted the data. JR-P wrote the manuscript. NM-D, AG, NG-V, and DB-E contributed to manuscript preparation. All authors contributed to the article and approved the submitted version.

## Funding

This work was supported by Universidad de Antioquia-CODI (Convocatoria Programática 2012–2013: Área de Ciencias Biomédicas y de la Salud, CPT-1217; and Convocatoria Programática 2017–2018: Área Ciencias de la Salud, CPT-1713) and Colciencias/Ministerio de Ciencia, Tecnología e Innovación de Colombia (Grants 1115-807-63108, 1115-569-33796, and 1115-519-29213).

## Conflict of Interest

The authors declare that the research was conducted in the absence of any commercial or financial relationships that could be construed as a potential conflict of interest.

## Publisher’s Note

All claims expressed in this article are solely those of the authors and do not necessarily represent those of their affiliated organizations, or those of the publisher, the editors and the reviewers. Any product that may be evaluated in this article, or claim that may be made by its manufacturer, is not guaranteed or endorsed by the publisher.

## Abbreviations

Ag: Antigen
BCA: Bicinchoninic acid assay
CD: Cluster of differentiation
CL: Cutaneous leishmaniasis
ConA: Concanavalin A
CpG: CpG Oligodeoxynucleotide
DC: Dendritic cell(s)
DisCL: Disseminated CL
dLN: Draining lymph node
ELISA: Enzyme-linked immunosorbent assay
GM-CSF: Granulocyte-macrophage Colony-stimulating factor
HRP: Horseradish Peroxidase
id: Intradermal
IFNγ: Interferon Gamma
Ig: Immunoglobulin
IL: Interleukin
ip: Intraperitoneal
*L(V)*: *Leishmania (Viannia)*
*L(V)b*: *Leishmania (Viannia) braziliensis*
*L(V)g*: *Leishmania (Viannia) guyanensis*
*L(V)p*: *Leishmania (Viannia) panamensis*
*L(L)m*: *Leishmania (Leishmania) major*
LCL: Localized CL
LN: Lymph node
mAb: Monoclonal antibody
ML: Mucosal leishmaniasis
MW: Molecular weight
NNN: Novy-MacNeal-Nicolle medium
PBMC: Peripheral blood mononuclear cell
PBS: Phosphate-buffered saline
PMA: Phorbol 12-myristate 13-acetate
RCL: Recurrent CL
SC: Subcutaneous
SDM: Schneider’s drosophila medium
SDS: Sodium dodecyl sulfate
SPF: Specific pathogen-free
TGFβ: Transforming growth factor beta
Th: T helper
TNFα: Tumor necrosis factor alpha
Tregs: Regulatory T cells
VEGF: Vascular endothelial growth factor
wpi: Weeks postinfection
