# Supplementary material for: A Mouse Model of Ulcerative Cutaneous Leishmaniasis by Leishmania (Viannia) panamensis to Investigate Infection, Pathogenesis, Immunity, and Therapeutics
Source: Front Microbiol. 2022 Jun 13;13:907631. doi: 10.3389/fmicb.2022.907631 (PMC9234518; doi:10.3389/fmicb.2022.907631)
Supplement: Supplementary file 1 [file Data_Sheet_1.PDF]

Supplementary Material

Supplementary Materials and Methods

Animals

BALB/c mice purchased to Charles River (USA) were bred and maintained in a SPF animal facility at the Sede de Investigación Universitaria (SIU), Universidad de Antioquia (UdeA). Female, 7-11 weeks old mice were used in all experiments. All *in vivo* procedures were approved by the institutional ethical animal committee.

Parasites and antigen

The UA-946 *L(V)p* stock (MHOM/CO/93/UA-946) was originally isolated at the Programa de Estudio y Control de Enfermedades Tropicales (PECET, UdeA, Medellin, Colombia) from a 24 years-old individual suffering LCL. Two separate ulcers with approximately 2 months of evolution were documented. The patient was successfully treated with antimonials (Glucantime® 20 mg/kg/day, 20 days). The species identity of this isolate was confirmed by using various methods (such as isoenzyme profiling, immunofluorescence microscopy with species-specific mAb and PCR-based assays with species-specific primers). Further, the genome sequence of the UA-946 *L(V)p* isolate has been reported (Urrea et al., 2018). After isolation, promastigotes were grown at 26°C in NNN or Schneider's Drosophila medium (SDM, Sigma, USA) supplemented with 10% heat-inactivated FCS (Gibco, USA), 2% filtered sterile human urine, 100 U/ml penicillin (Gibco, USA), and 100 µg/ml streptomycin (Gibco, USA). Five to six-day cultures (early stationary phase) were used. The UA-946 *L(V)p* strain was adapted to grow in mouse tissues by long-term serial maintenance in BALB/c mice, as follows: 10^6 stationary promastigotes were injected in the footpads and 2-6 months later a new isolate was obtained from these mice to repeat the process for at least three more passages *in vivo*. After this adapting procedure, most of infected mice presented swelling in the footpads, and also ulcers could be induced in animals infected in the base of the tail or the ear. *Leishmania (Leishmania) major* (*L(L)m*; MHOM/IL/81/BNI, kindly provided from Prof. H. Moll, Institute for Molecular Infection Biology, University of Würzburg, Germany (Ramírez-Pineda et al., 2004)), was used in some experiments as a control. *L(L)m* was maintained *in vivo* and *in vitro* by periodical passages in BALB/c mice and by using NNN medium, respectively.

Total *Leishmania* antigen (Ag) from both *L(V)p* and *L(L)m* were prepared by resuspending in PBS 1×10^9 parasites/ml from early stationary promastigote cultures and performing 10 cycles of freezing (below -80°C)/thawing-sonication to obtain a lysate suspension. For some experiments, total *L(V)p* lysate was fractionated using an electroelution device (Whole Gel Eluter, Cat. 165-1251, BioRad). Alternatively, a manual fractionation strategy was implemented that allowed a better yield of fractions for *in vivo* experiments. Briefly, 500 µg of total *L(V)p* lysate were run on a preparative 12 % SDS-PAGE at 150 V for 5 hours using a ProteanXi system (BioRad). After electrophoresis, the gel was cut into horizontal strips (spaced from one another by about 0.5 to 1 cm), starting from the bottom of the gel. Then, each strip was separated into smaller pieces, finely homogenized, and placed in 0.5 ml of endotoxin-free PBS. The resulting dispersion was stored overnight at 4°C to allow protein diffusion, and dialysis was performed to remove the remaining SDS. Protein concentration in total Ag and fractions was determined by the BCA method (Pierce, USA), and stocks were stored at -80ºC until use.

Infection, clinical follow-up, sample obtention and parasitological evaluation

Animals were infected into the right hind footpad (subcutaneously, sc), the base of the tail (sc) or the right ear (intradermally, id) with 10^6 (in 50 µl), 10^5 (in 50 µl) or 10^5 (in 20 µl) stationary promastigotes, respectively.

A clinical follow-up was performed consisting in the assessment of body weight and lesion development. The footpad thickness and lesion size were registered weekly with the help of a digital caliper (precision: 0.02 mm, model MT-00855, Uyustools, China). The footpad swelling was calculated as the difference in the thickness (in mm) between the infected and the contralateral non-infected foot. Lesion size in the base of the tail and the ear was reported as the area (in mm^2) by measuring the two crossed diameters of lesions and calculating with the formula $A = \pi\left(\frac{D1+D2}{4}\right)^2$. At different timepoints after infection, mice were euthanized to obtain tissues for parasitological, histopathological, and immunological analysis. Infected tissues were removed, weighted, and cultured to determine the parasite burden per organ (footpad and ear) or mg of tissue (base of the tail) by using a limiting dilution protocol (Buffet et al., 1995; Lima et al., 1997) with minor modifications. Cell suspensions from dLN and spleens were also seeded in supplemented SDM and incubated at 26° C to determine the presence of viable amastigotes in those tissues.

Pharmacological and immunomodulatory interventions

In experiments designed to test therapeutic agents in the model, infected mice received antimonial treatment (a weekly intraperitoneal injection of Glucantime®, 500 mg/kg, for 4 weeks), or Miltefosine (20 mg/kg/day, orally for five consecutive days), administered after disease establishment (4-5 weeks postinfection). Lesion progression was monitored as above and once clinical cure was observed, mice were euthanized to determine parasite burdens. No clinical toxicity was observed at these doses, as assessed by changes in the animals' body weight or general appearance (not shown). In other experiments, mice were infected as above, but additionally co-injected with synthetic CpG-containing oligodeoxynucleotide 1826 (CpG; 5'-TCCATGACGTTCCTGACGTT-3'; phosphorothioate modified; Integrated DNA Technologies - IDT, USA). The effect of CpG, Ag or Ag+CpG administered (two or four weeks) prior to infection was also assessed in vaccination experiments. In additional vaccination experiments, 5-12 µg of different fractions of the parasite lysate were administered in combination with CpG following the scheme of two vaccine doses (separated by two weeks) and an infective challenge four weeks after the boost. Mice that were protected from *L(V)p* infection as a result of previous vaccination with the fraction F9+CpG, received a secondary challenge at the 11th week post primary infection and followed-up.

Cytokine secretion by dLN cells and splenocytes

Draining lymph nodes (dLN) and/or spleens were aseptically removed, and their weight individually determined. Tissue was mechanically disrupted with 70-µm nylon cell strainers (Corning, USA) and total cell counts were determined. A previous step of red blood cell lysis was included in spleens samples. Cell suspensions were prepared at 2×10^6 cells/ml in culture medium (RPMI 1640-GlutaMAX, Gibco, USA) supplemented with 10% FCS, 100 U/ml penicillin, and 100 µg/ml streptomycin. One ml cell suspensions were dispensed in 24-well plates and stimulated with 10 µg *L(V)p* Ag for 72 hours. Untreated or Concanavalin A (ConA, 5 µg/ml)-stimulated cultures were included as negative and positive controls, respectively. Culture supernatants were next collected to determine the concentration of cytokines by using ELISA kits (Opt EIA kits, BD, USA; R&D systems, USA) or 12-plex or 32-plex multi-analyte Luminex technology kits (Bioplex, Bio-Rad, USA). The quantification ranges were 3.2-10000 pg/ml for Luminex and 7,8-500 pg/ml, 31.3-2000 pg/ml, 62.5-4000 pg/ml, 3.1-200 pg/ml for ELISA IL-4, IL-10, IL-13, and IFNγ, respectively. Cells were always responsive, as confirmed by the cytokines secreted in response to ConA stimulation; in cell suspensions from naive mice, cytokine response to *L(V)p* Ag was negligible (not shown).

L(V)p-specific antibody measurement

Blood samples were centrifuged for 20 minutes to obtain the serum, which was subsequently stored at -20°C until further use. The levels of *L(V)p*-specific immunoglobulins (Ig) IgG1 and IgG2a were measured by ELISA (Ramírez-Pineda et al., 2004). Antibody binding was revealed with a specific secondary antibody for each isotype (biotinylated anti-mouse IgG1 clone A85-1, and biotinylated anti-mouse IgG2a clone R19-15; BD, USA) followed by streptavidin-HRP (BD, USA). The reaction was developed with tetramethylbenzidine (TMB) substrate (BD, USA), and the absorbance spectrophotometrically determined at 405 nm (PowerWave BioTek®) or 655nm (iMark, Bio-Rad).

Histopathology

After euthanasia, footpads and ears were removed and fixed with 10% buffered formaldehyde. 24 hours later, organs were routinely processed for paraffin embedding (a previous decalcification step with nitric acid was included for foot samples). Tissue sections (5 µm thick) were stained with hematoxylin/eosin and analyzed by light microscopy.

Leukocyte isolation from ear tissue

Infected and control ear tissues were aseptically removed from mice and the isolation of inflammatory cells was achieved according to the literature (Duthie et al., 2007; Anderson et al., 2008). Briefly, ears were treated with PBS supplemented with penicillin (300 U/ml) and streptomycin (300 µg/ml) for 30 minutes at 37°C. Then, ear tissue was split into dorsal and ventral sheets and the inflammatory infiltrate was removed and cut in small pieces which were transferred to 1 ml PBS supplemented with 0.5 mg/ml Liberase TL Research Grade (Roche Diagnostics, Switzerland) for 1 hour at 37°C. Digested ears were manually homogenized in 70-µm nylon cell strainers (Corning, USA) with the help of a syringe plunger. The resultant homogenate was washed twice with PBS at 550×*g* for 5 minutes and suspended in supplemented RPMI 1640-GlutaMAX. Finally, the suspension was mixed up and down 5 times in a syringe to disrupt the remaining agglomerations, filtered through a 70-µm nylon cell strainer (Corning, USA), washed again, and suspended in supplemented RPMI 1640-GlutaMAX to be counted. Free cells were resting overnight at 37ºC, 5%CO_2.

Flow cytometry staining and analysis

Leukocytes from dLN and ear tissue were surface stained with CD3e FITC (145-2C11, BD, USA), CD4 PE (GK1.5 BD) or PECy5 (GK1.5 eBioscience, USA), CD8 PECy5 (53-6.7 eBioscience), CD11c PECy7 (HL3, BD), CD11b PE (M1/70, BD) and Gr1 FITC (RB6-8C5, BD Biosciences). Staining was performed following the manufacturer’s indications after CD16/32 Fc blockage (2.4G2, BD) of pooled dLN or ear cell suspensions. To intracellular staining, cells were incubated during 6 hours with PMA/Ionomycin and Monensin (eBioscience, USA) under culture conditions. Cells were then collected, washed, and surface stained as described above, followed by intracellular staining with IFNγ PE (XMG1.2 BD) and IL-4 PE (11B11, BD) or their counterpart isotypes as controls. Samples were acquired with a FACSCanto™ II flow cytometer (BD, USA) and analyzed using the FACSDiva™ 6.1 software (BD, USA) following the gating strategy described in **Supplementary Figure S6**. The viabilities of dLN and ear tissue cell suspensions were always greater than 99% and 93%, respectively, as assessed by DiOC6 staining.

Data analysis

Data were tabulated, plotted, and analyzed using GraphPad Prism 8.3 (GraphPad Software Inc., San Diego, CA, USA). Results are presented in figures as the median, median ± interquartile ranges or ranges, geometric mean, or geometric mean ± 95%CI with/without representing individual mice as dots or lines. In some graphs, data are presented using a "box and whiskers" plot in which box hinges ranged from the 25th to the 75th percentile and the median (line), mean (+) and min/max data (whiskers) are represented. For the comparison of two unpaired groups, the Mann-Whitney *U* test was used, and for the comparison of more than two unpaired groups (control versus each treatment), the nonparametric Kruskal-Wallis test with a Dunn's post-test was performed. To analyze multiple comparisons through time (repeated measures) we employed a mixed-effects analysis or a two-way ANOVA with Geisser-Greenhouse correction and Bonferroni's multiple comparisons post-hoc test. To assess the relationship between parasite burden and lesion size, a Spearman rank correlation analysis was carried out. When a particular statistical test is performed, it is specified in the corresponding legend. $p<0.05$ was considered significant (*$p<0.05$; **$p<0.01$; ***$p<0.001$; ****$p<0.0001$).

Supplementary Tables

Supplementary Table S1. ACL patients and *L(V)p* isolates.

Patient code (Clinical form)	Place of origin (Province)	Age In years	IDR	Number of lesions	Time of Evolution In months
**UA-946 (LCL)**	Dabeiba (Antioquia)	24	+	2	2
**UA-1114 (LCL)**	La Victoria (Caldas)	29	+	1	3
**UA-873 (LCL)**	Puerto Valdivia (Antioquia)	28	+	2	3
**UA-936 (ML)**	San Pedro de Urabá (Antioquia)	26	+	1	U
**UA-1472 (ML)**	Herveo (Tolima)	52	+	1	4
**UA-1511 (ML)**	Puerto Valdivia (Antioquia)	23	+	1	1
**UA-1756 (DisCL)**	Caimán Nuevo (Antioquia)	43	+	50	1.5
**UA-1086 (DisCL)**	La Victoria (Caldas)	24	+	28	1
**UA-1446 (DisCL)**	Puerto Valdivia (Antioquia)	20	+	15	1.5
**UA-1759 (RCL)**	Mutatá (Antioquia)	28	+	1	1*
**UA-1741 (RCL)**	Puerto Valdivia (Antioquia)	40	+	1	2*
**UA-1003 (RCL)**	La Victoria (Caldas)	50	+	6	1*

Clinical-epidemiological information about patients and *L(V)p* isolates is presented. Colombian patients were diagnosed with CL at the Programa de Estudio y Control de Enfermedades Tropicales (PECET), Universidad de Antioquia, Medellín, Colombia. The intradermal reaction (IDR), as assessed by the Montenegro skin test, was considered positive when greater that 5 mm. LCL: localized cutaneous leishmaniasis, ML: mucosal leishmaniasis, DisCL: disseminated cutaneous leishmaniasis and RCL: recurrent cutaneous leishmaniasis. For LCL, ML and DisCL cases, no patient had previous antecedent of ACL or received specific antileishmanial treatment at the time of diagnosis and parasite isolation. RCL were patients that had primary LCL lesions that were successfully treated with complete supervised healing, but reactivation was observed in the scares. In these cases, the time of evolution of reactivation is presented (*). Microsatellite typing of paired isolates (primary and secondary lesions) indicated that recurrence was due to reactivation instead of new infection. U: unknown. Species identification for all isolates was performed by isoenzyme, mAb and/or molecular methods.

Supplementary Table S2. ***L(V)p*** **viable parasites persist in infected tissues from BALB/c mice.**

**Species / strain code**	**3 months**				**8 months**			
Culture from:	Footpad		dLN		Footpad		dLN	
Inoculum:	10^6	10^7	10^6	10^7	10^6	10^7	10^6	10^7
L. panamensis MHOM/CO/93/UA-946	+/+	+/+	+/+	+/+	+/+	+/+	+/+	+/+
L. panamensis MHOM/CO/93/UA-1114	+/+	+/+	c/+	+/+	+/+	+/+	+/+	+/+
L. panamensis MHOM/CO/93/UA-873	+/+	+/-	+/+	+/-	Nd	Nd	Nd	Nd
L. panamensis MHOM/CO/93/UA-936	-/-	-/-	c/-	-/-	Nd	Nd	Nd	Nd
L. panamensis MHOM/CO/94/UA-1472	-/-	-/-	-/+	-/-	Nd	Nd	Nd	Nd
L. panamensis MHOM/CO/97/UA-1511	+/c	-/-	-/c	-/-	+/+	+/+	+/+	+/+
L. panamensis MHOM/CO/99/UA-1756	-/-	-/-	-/-	-/-	Nd	Nd	Nd	Nd
L. panamensis MHOM/CO/93/UA-1086	+/+	-/-	+/+	-/-	Nd	Nd	Nd	Nd
L. panamensis MHOM/CO/96/UA-1446	+/+	+/-	+/+	-/-	Nd	Nd	Nd	Nd
L. panamensis MHOM/CO/99/UA-1759	+/+	-/+	+/+	+/+	Nd	Nd	Nd	Nd
L. panamensis MHOM/CO/99/UA-1741	+/+	-/-	+/+	+/+	Nd	Nd	Nd	Nd
L. panamensis MHOM/CO/93/UA-1003	+/+	-/-	+/+	-/+	Nd	Nd	Nd	Nd
L. major MHOM/IL/81/FE/BNI	+/+	+/+	+/+	+/+	Nd	Nd	Nd	Nd

Footpad aspirate samples from mice that were infected with 10^6 or 10^7 parasites (**Supplementary Figure S1**) were cultured to determine the presence of viable parasites at 3 months after infection. Parallel parasite cultures of draining lymph node (dLN) cell suspensions were also set up. Two mice per experimental condition were used and analyzed individually. The presence (+) or absence (-) of motile promastigotes was reported. Note that viable parasites could be recovered from mice infected with most of *L(V)p* isolates. Samples from spleen and liver tested positive in *L. major*-infected but not in *L(V)p*-infected mice. In an independent similar experiment with the isolates UA-946, UA-1114 and UA-1511, parasites could be also recovered from the footpads and dLN of all infected mice 8 months after infection. c: fungal/bacterial contamination in parasite cultures. Nd: not determined.

Supplementary Table S3. Antigen-specific cytokine and growth factor response in the dLN and the spleen of *L(V)p*-infected BALB/c mice. An Excel workbook containing raw data from Luminex analysis is presented as supporting information for **Figure 4A** and **Supplementary Figure S8.** Quantification ranges of each analyte are presented in a separated sheet. Cells highlighted in yellow correspond to those concentrations calculated from the regression curves in which the luminescence value lies below the lower limit of quantification. Data are presented in pg/ml.

Supplementary Figures

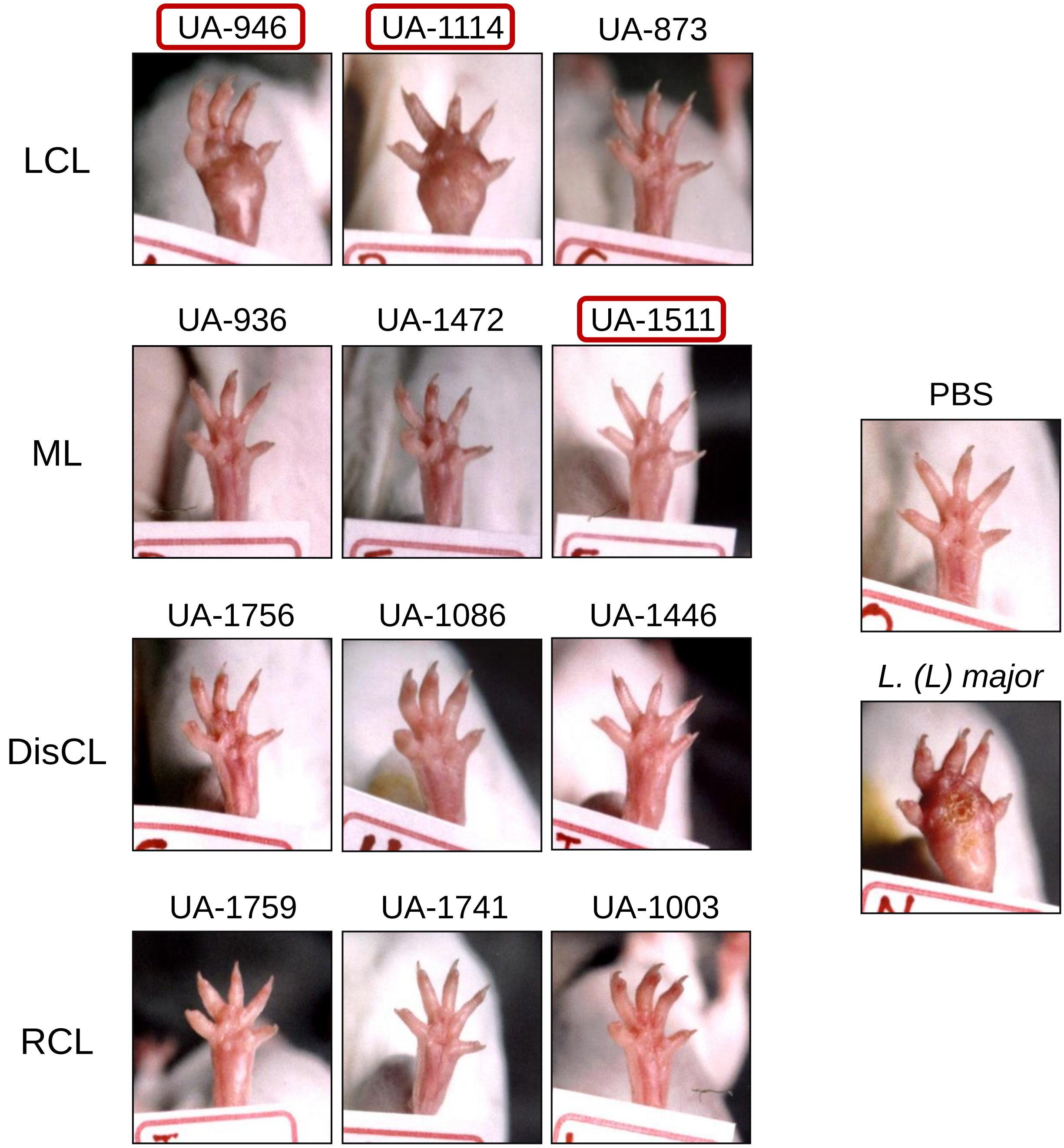

Supplementary Figure S1. Infection of BALB/c mice with human *L(V)p* isolates. BALB/c mice were infected sc in the footpad with 10^7 stationary promastigotes of the indicated human *L(V)p* isolate (see **Supplementary Table S1** for details). Mice injected with PBS or *L(L)m* (strain BNI; 10^5 stationary promastigotes) were used as negative and positive controls, respectively. Mice were followed weekly to monitor lesion development. Representative photographs from experimental groups (n=2) at 7th week postinfection are shown. Whereas infections with most of *L(V)p* isolates were asymptomatic and footpads looked like non-infected mice during the whole observation period, the isolates UA-946, UA-1114 and UA-1511 induced non-ulcerative mild inflammation (red squares). As expected, mice infected with *L(L)m* developed ulcerative inflammatory progressive lesions.

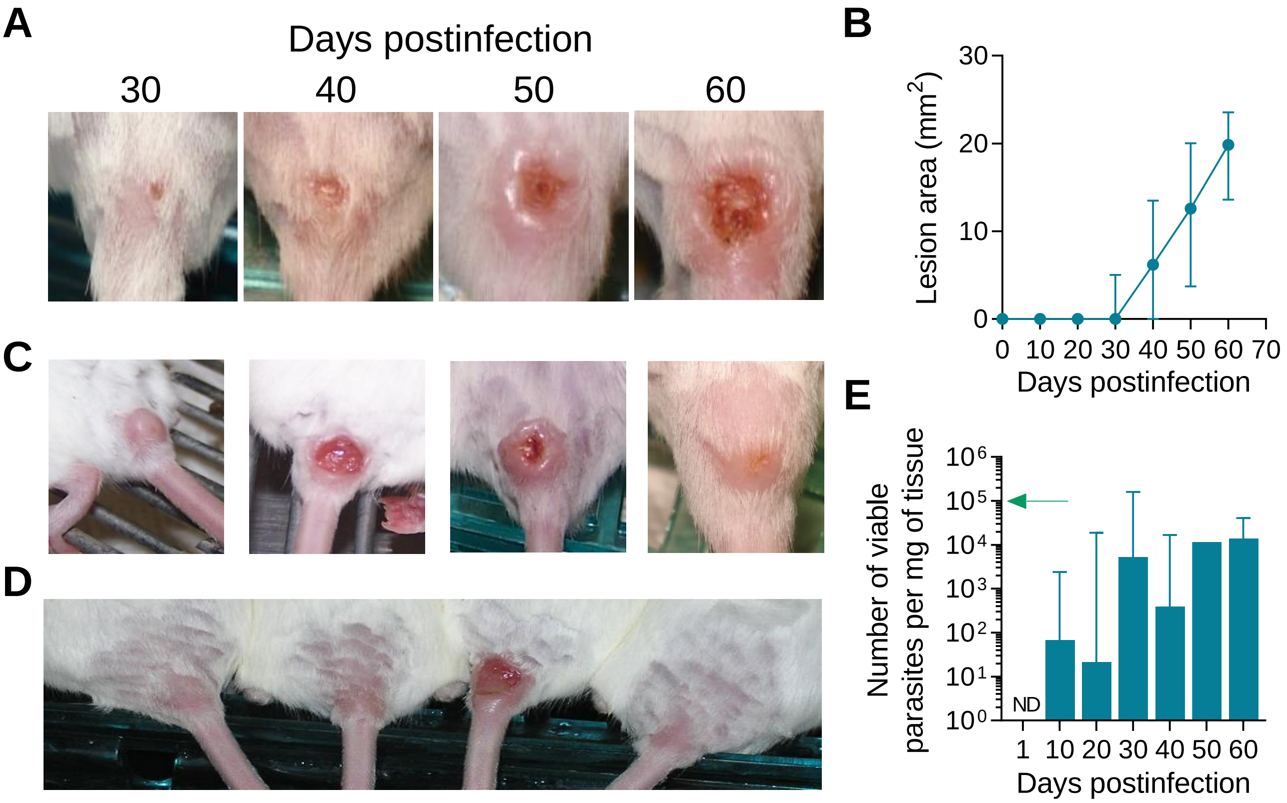

Supplementary Figure S2. Clinical and parasitological characterization of BALB/c mice infected with *L(V)p* in the base of the tail. BALB/c mice were infected sc with 10^5 *L(V)p* stationary promastigotes in the base of the tail and monitored. Representative photographs at the indicated time postinfection are presented **(A)**. The area of the lesions was individually measured and graphed **(B)**. It was common to observe a great heterogeneity in the size and appearance of the lesions within and among experiments, as documented with some photographs of mice infected for 8-9 weeks in independent experiments **(C)**. Moreover, note how in a separate experiment only a minor fraction of infected mice exhibited measurable lesions with most of mice being asymptomatic **(D)**, which contrasted with a parallel cohort of BALB/c mice infected with *L(L)m* in the same anatomical location (100% of animals exhibiting progressive ulcers, not shown). At the indicated time, mice from **A-B** were sacrificed, and the number of viable parasites determined and normalized per mg of tissue **(E)**. Data are presented as the median ± interquartile range **(B,** n=6**)** and geometric mean ± 95%CI **(E,** n=1-3**)**. The arrow in **(E)** indicates the infective inoculum. ND: not detected.

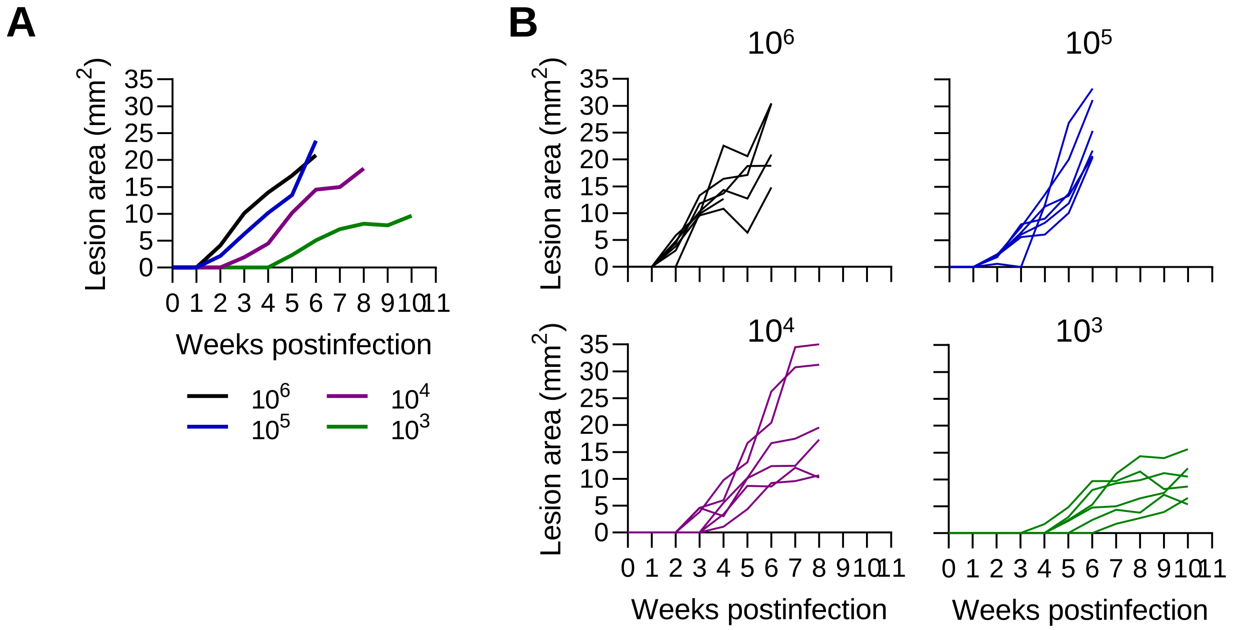

Supplementary Figure S3. Variation in the kinetics of lesion development as a function of the size of the inoculum. BALB/c mice were infected in the ear dermis with the indicated inoculum of UA-946 *L(V)p* promastigotes and the disease progression monitored weekly. Lesion sizes were graphed as the median (**A**) or individual mice (**B**). Note the prolongation of the incubation period and the slower progression of lesions as inoculum size is reduced.

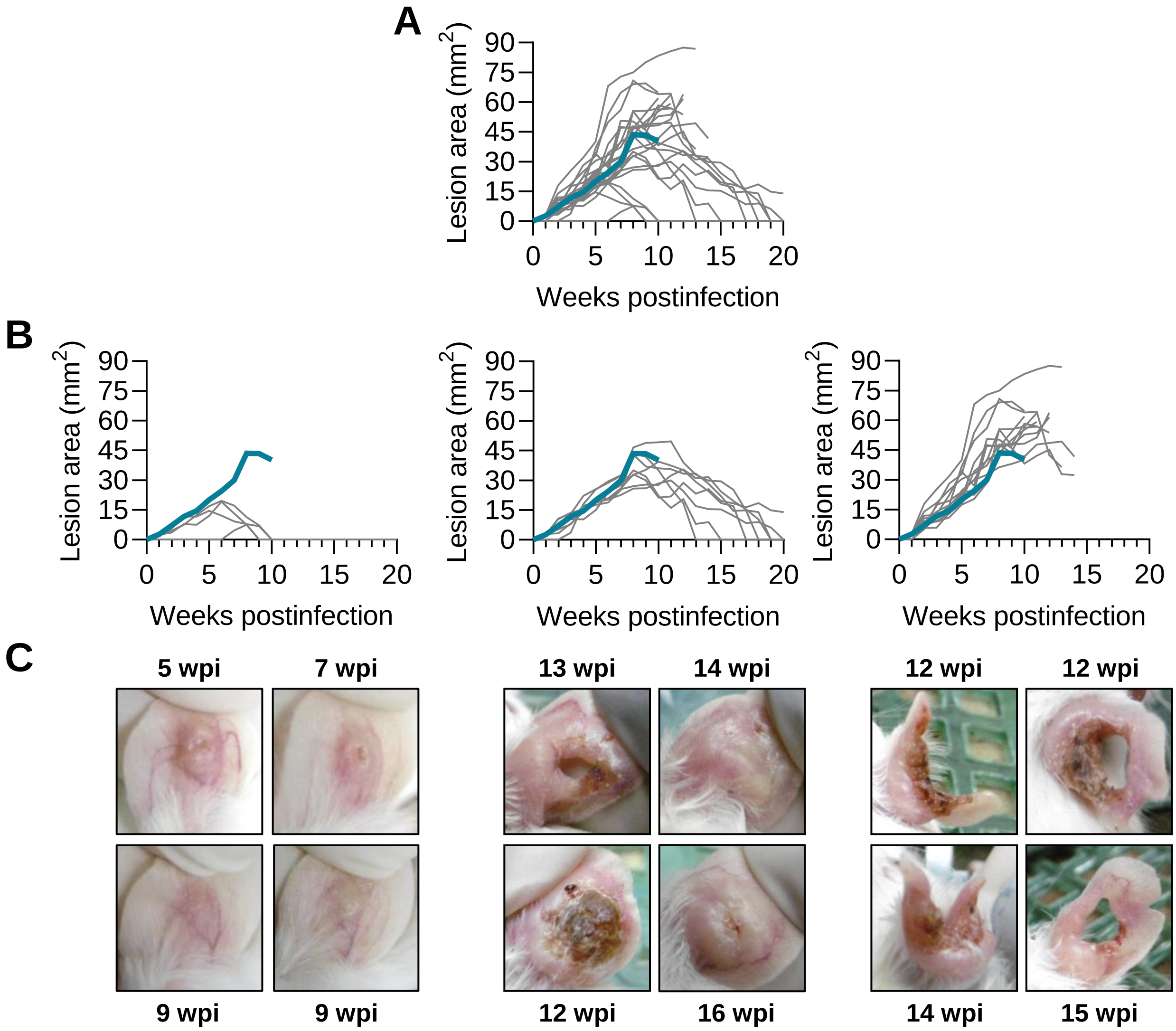

Supplementary Figure S4. Extended follow-up of infected ears confirms the chronic, tissue damaging and slow resolving nature of the *L(V)p*-BALB/c model. BALB/c mice (n=25) were infected with 10^5 *L(V)p* stationary promastigotes intradermally in the right ear and monitored weekly. Lesion areas were determined individually for an extended period of 20 weeks postinfection and graphed as the median [thick line] and for each individual mouse [thin lines] (**A**). Since partial or total mutilations of the ears began to be observed from week 11 postinfection precluding further lesion measurement, average lesion is only graphed up to week 10. As previously noted (**Figure 2**), a variety of clinical outcomes upon infection and significant heterogeneity is observed, particularly after week 5-6 **(A)**. The three categories that can be established depending on the magnitude/severity and duration of the resulting disease are graphed separately **(B)**. The first minor group consists of animals exhibiting a fairly asymptomatic infection or clinical development of small lesions peaking at week 4-7 postinfection but resolving immediately within 10 weeks postinfection **(B,** *left***)**. Remaining (majority) animals exhibit prolonged large lesions that follow one of two outcomes: i) slow-resolving chronic lesions that usually cure within 20 weeks postinfection without loss of the organ **(B,** *center***)**, and ii) very chronic tissue-destructive lesions ending with partial or total mutilation of the ear **(B,** *right***)**. Mutilation often impedes continuation of lesion size measurements resulting in incomplete quantitative monitoring (interrupted lines in **B,** *right*). Further qualitative follow-up indicated that most of animals in this group completely resolve the lesions at week 20-22 postinfection. Independent experiments, however, have shown that complete healing can take up to 24 weeks (not shown). Representative photographs of animals belonging to each group at the indicated time postinfection are presented **(C)**. wpi: weeks postinfection.

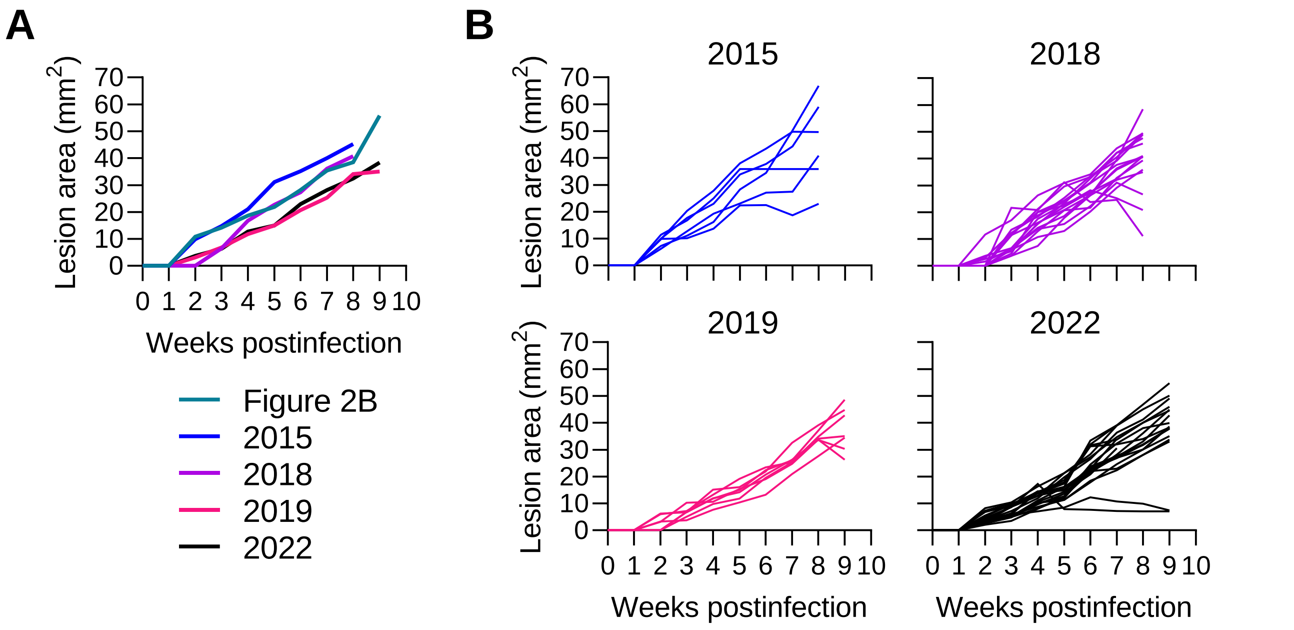

Supplementary Figure S5. The UA-946 *L(V)p* ulcerative model is highly reproducible. Data from the non-treated control groups used in four independent interventional experiments performed in the indicated year were graphed as the median (**A**) or individual mice (**B**). Median of lesion size from **Figure 2B** is also shown in **(A)** for comparison.

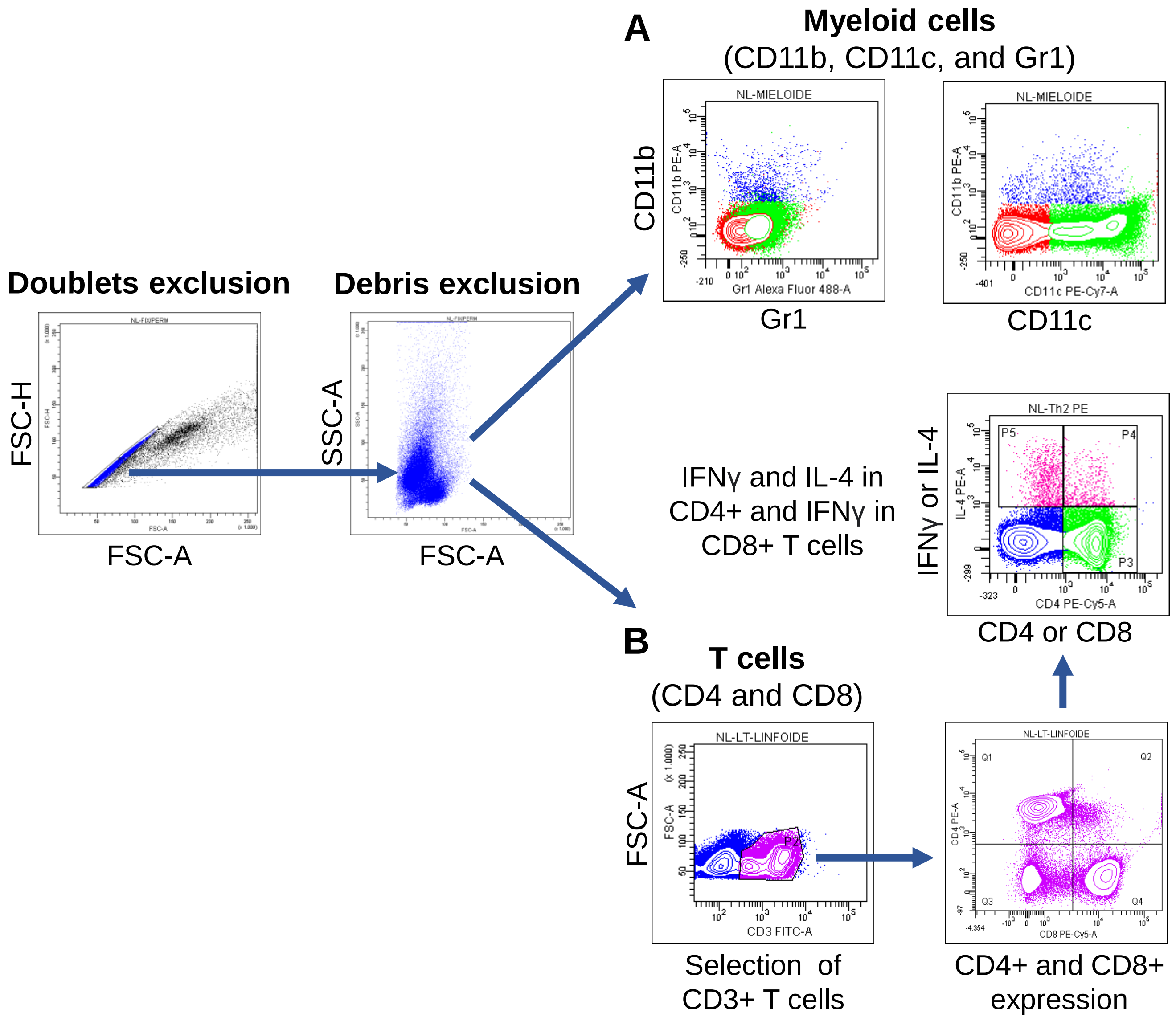

Supplementary Figure S6. Flow cytometry gating strategy to evaluate myeloid cells and T cells in the dLN and the ears of *L(V)p*-infected BALB/c mice. Cell suspensions from the dLN were acquired and cleaned by doublets exclusion and selecting populations without debris. Selected cells were discriminated into myeloid cell subpopulations on the basis of the expression of CD11b, CD11c and Gr1 markers **(A)**. Also, CD3+ CD4+ or CD3+ CD8+ T cells were quantified, which were further analyzed on the basis of cytokine expression as IFNγ-producing or IL-4-producing T cells **(B)**. A similar strategy was applied with cell suspensions prepared from pooled ear tissue, with exception that the gating of CD3+ cells was replaced by the selection of the lymphoid cell region in the forward/side scattering plot, given the loss of surface CD3 expression in tissular T cells from infected mice. All populations were defined according to isotype controls for each data set.

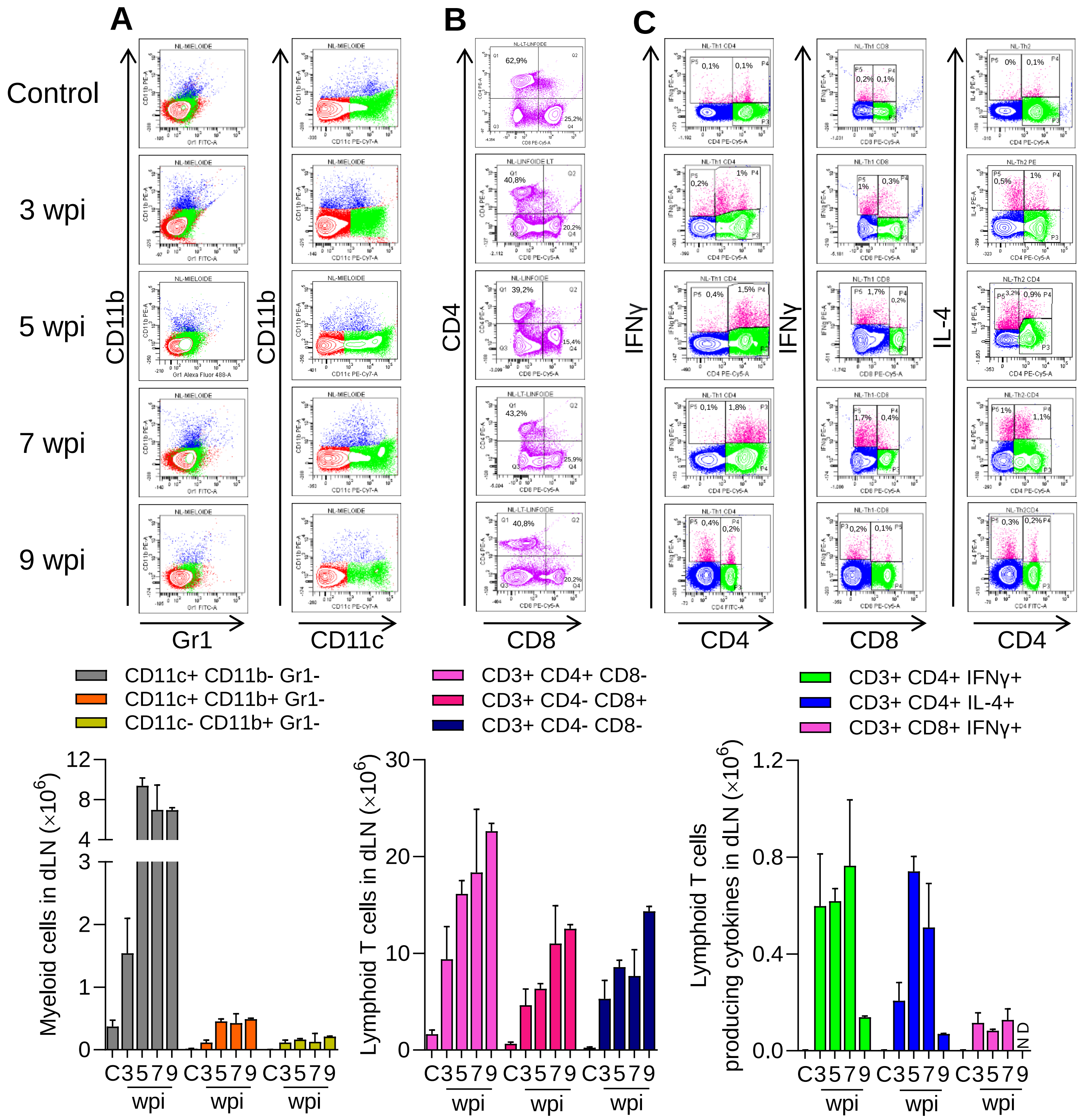

Supplementary figure S7. Increasing numbers of myeloid cells and IFNγ or IL-4-expressing T lymphocytes in the dLN of *L(V)p*-infected mice. Mice were infected with *L(V)p* and sacrificed at the indicated time point to obtain cell suspensions from the dLN in order to analyze different leukocyte populations by flow cytometry following the gating strategy explained in **Supplementary Figure S6.** Flow cytometry acquisition was performed from samples pooled by time of sacrifice. The surface expression of CD11b, CD11c, and Gr1 markers allowed the identification of three myeloid cell subpopulations: CD11c+ CD11b- Gr1-, CD11c- CD11b+ Gr1- and CD11c+ CD11b+ Gr1- **(A)**. The surface expression of CD3, CD4, and CD8 markers allowed the quantification of the subpopulations CD4+, CD8+ and CD4-CD8- double negative (DN) T cells **(B).** Cells were also analyzed for the intracellular expression of IFNγ and IL-4 after PMA/ionomycin stimulation as CD4+ IFNγ+, CD4+ IL-4+ and CD8+ IFNγ+ T cells **(C)**. Representative contour plots (upper panel) and the absolute numbers of the indicated cell subpopulation per dLN (lower panel) are shown. Non-infected mice were used as controls for comparison. Data are shown as median ± interquartile range (n=3-5 mice). wpi: weeks postinfection. C: control. ND: not detected.

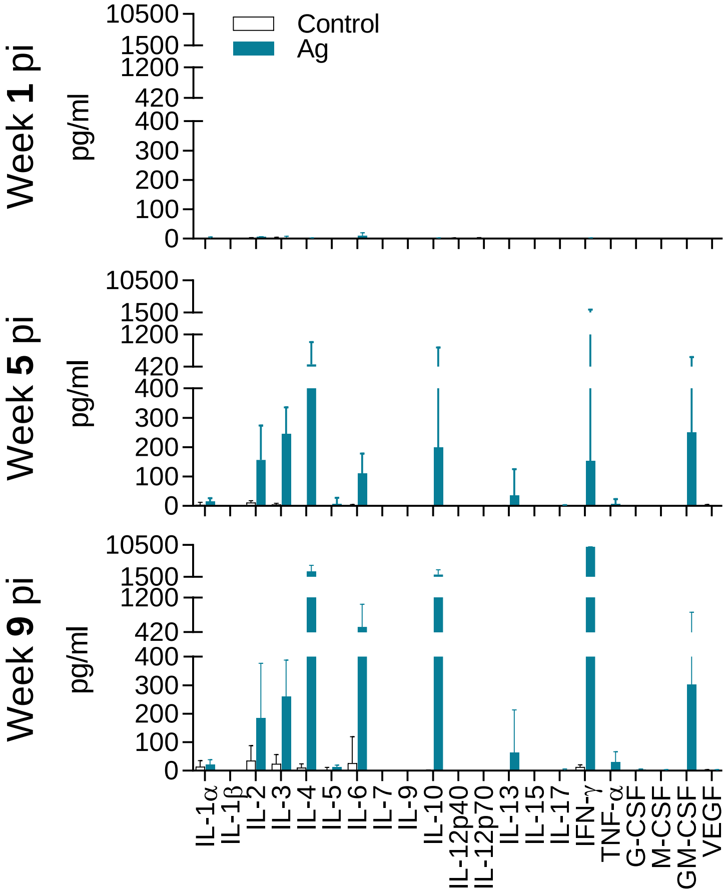

Supplementary Figure S8. Antigen-specific cytokine response in the spleen of *L(V)p*-infected BALB/c mice. Spleens from mice of **Figure 4** were macerated to obtain cell suspensions. Cells from mice infected at the indicated timepoint were cultured in the absence (Control) or presence (Ag) of *L(V)p* total lysate. The concentration of the indicated cytokine in supernatants was determined by a multi-analyte Luminex platform and graphed (as the median ± interquartile range). n=3-5 mice/group. pi: postinfection.

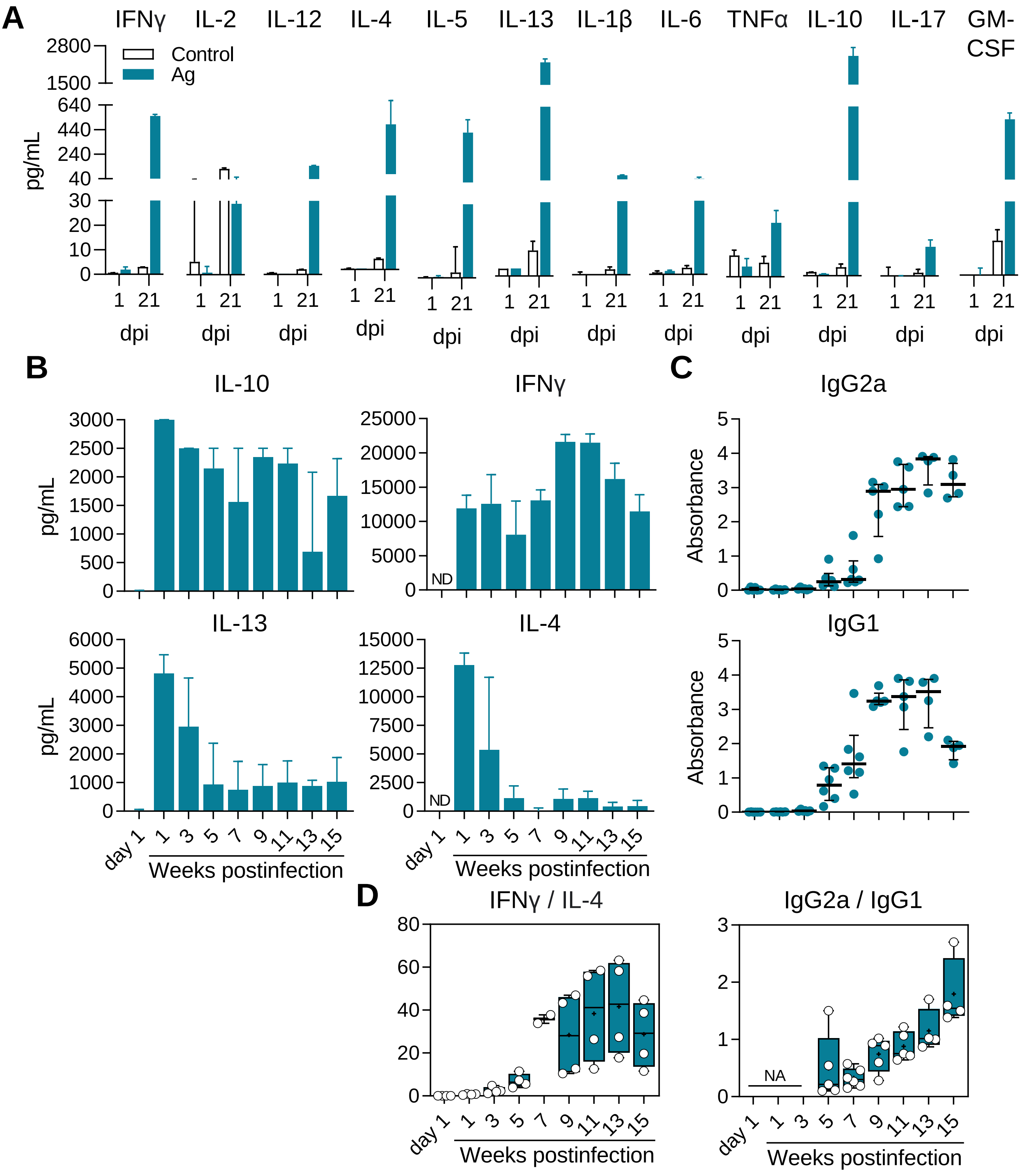

Supplementary Figure S9. Antigen-specific immune response in BALB/c mice infected with *L(V)p* in the footpad. BALB/c mice were infected sc with 10^6 *L(V)p* stationary promastigotes in the right footpad as in **Figure 1** and sacrificed at 1 or 21 days postinfection (dpi). dLN were macerated to obtain single cell suspensions that were stimulated with Ag or PBS as controls, and the concentration of the indicated cytokine secreted to supernatants determined by Luminex **(A).** The kinetics of the Ag-induced production of the cytokines IL-10, IL-4, IL-13, and IFNγ was also determined by ELISA in dLN suspensions of mice sacrificed at the indicated timepoints **(B)**. Serum samples from same animals were also used to quantify the levels of *L(V)p*-specific IgG1 and IgG2a antibodies by ELISA **(C)**. The IFNγ/IL-4 and IgG2a/IgG1 ratios were calculated and graphed as "box and whiskers" plots **(D)**. Data are presented as the median ± range or interquartile range (n=3-6 mice in **(A-C)** and 2-6 in **(D)**). ND: not detected. NA: not applicable.

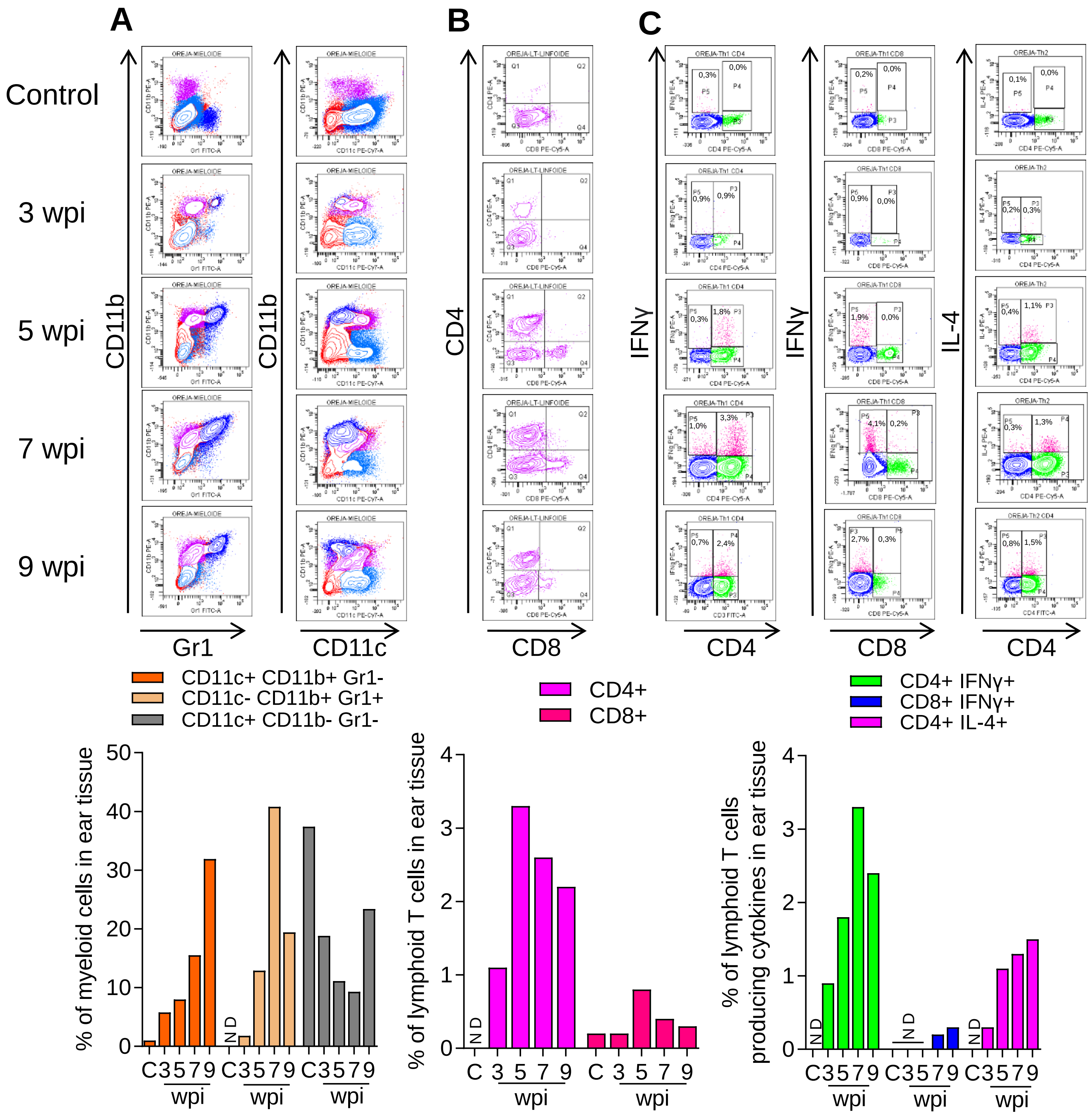

(see legend in following page)

Supplementary Figure S10. Infiltration of myeloid and cytokine-producing T cells to the site of *L(V)p* infection in BALB/c mice. Mice were infected in the ear with *L(V)p* and sacrificed at the indicated timepoint to obtain cell suspensions from pooled ears (n=3-5) in order to analyze different leukocyte populations by flow cytometry following the gating strategy explained in **Supplementary Figure S6**. The surface expression of CD11b, CD11c, and Gr1 markers allowed the identification of three myeloid cell subpopulations: CD11c+ CD11b+ Gr1-, CD11c- CD11b+ Gr1+, and CD11c+ CD11b- Gr1- **(A)**. CD4+ and CD8+ lymphocytes were also quantified **(B).** Cells were also analyzed for the intracellular expression of IFNγ and IL-4 after PMA/ionomycin stimulation as CD4+ IFNγ+, CD4+ IL-4+, and CD8+ IFNγ+ T cells **(C)**. Representative contour plots (*upper panel*) and the percentages of the indicated cell subpopulation per ear pool (*lower panel*) are shown. Non-infected mice were used as controls for comparison. Note that a CD11c- CD11b+ Gr1+ cell population, most likely corresponding to neutrophils, that was absent in non-infected ears, rapidly increased to represent 1.8%, 12.9% and 40.8% of recovered cells at weeks 3, 5, and 7 postinfection, respectively. When the control of parasite replication was evident and resolution of lesions in some animals began at week 9 postinfection, the frequency of these cells dropped to 19.4% and no longer represented the dominant population. A second major myeloid population (CD11c+ CD11b+ Gr1- cells) was present with increasing frequencies along disease development and represented 31.9% of the cell infiltrate in infected animals at the latest time point analyzed, compared to only 1.0% in uninfected ears. Conversely, CD11c+ CD11b- Gr1- cells, compatible with resident dendritic cells and present at a frequency of 37.4% in non-infected ears, rapidly decreased after infection to represent 18.8%, 11.1% and 9.3% at weeks 3, 5, and 7 postinfection, respectively, and to partially repopulate the tissue at week 9 postinfection (up to 23.4%). Note that T cells also appeared in infected ears as soon as the 3rd week postinfection and remained present for the rest of observation period. Both CD4+ and CD8+ T cells peaked at the 5th week postinfection to subsequently decline at 7th and 9th week postinfection. Increasing numbers of IFNγ-producing T cells were also detected in infected ears, mainly CD4+ peaking at week 7 postinfection and declining at week 9 postinfection and CD8+ representing a minor part. IL-4-producing CD4+ T cells were also present from the 1st to 9th week postinfection. C: control. ND: not detected. wpi: weeks postinfection.

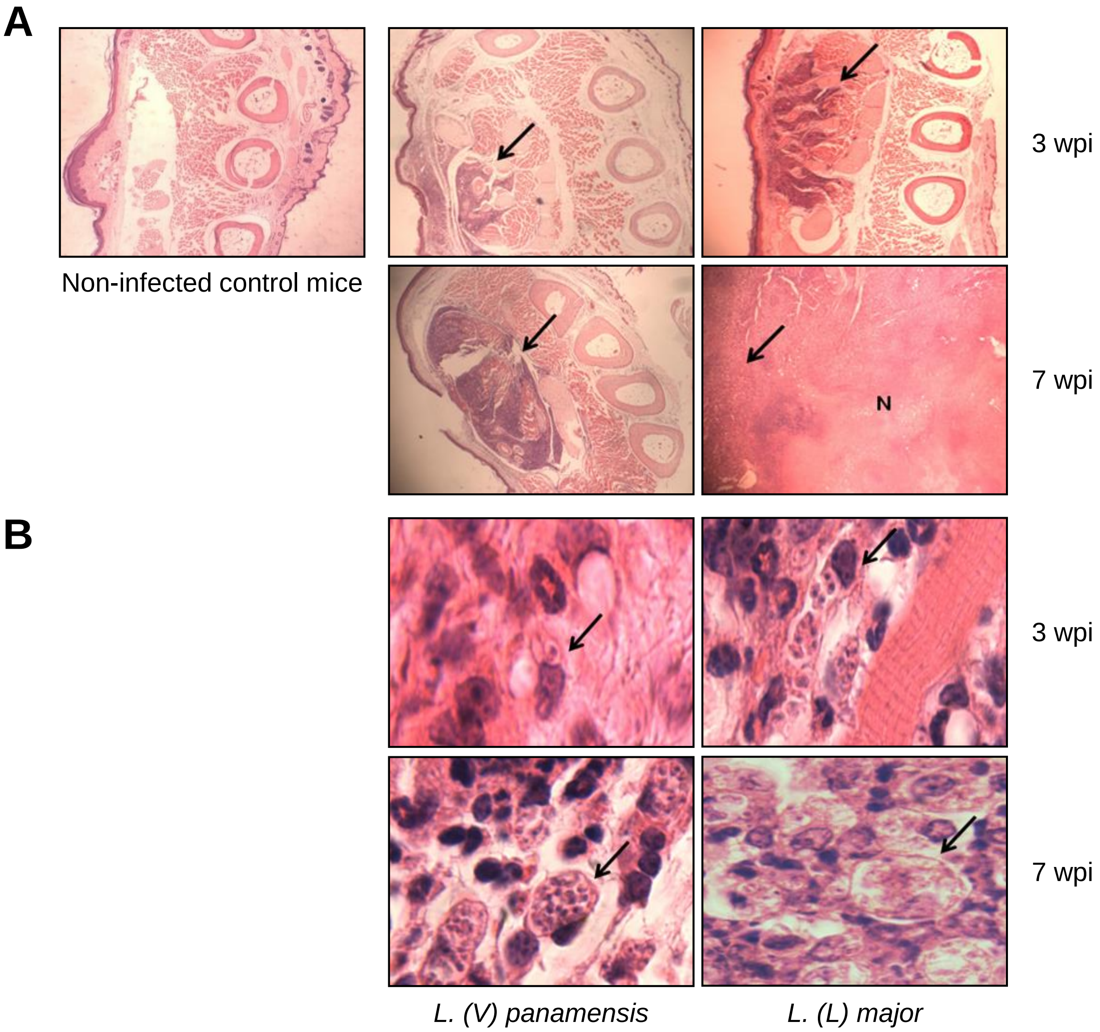

Supplementary Figure S11. Histopathological appearance of mice footpads infected with *L(V)p* or *L(L)m*. Footpads from mice of the experiment shown in **Figure 1** were obtained at the 3rd or 7th week postinfection and sections stained with hematoxylin/eosin. Representative photographs taken with low (4X) **(A)**, or high (100X) **(B)** magnification are shown. A section from a non-infected control footpad is shown for comparison **(A**, *left***)**. N: necrosis. wpi: week postinfection. Arrows in **(A)** indicate inflammatory infiltrate while arrows in **(B)** indicate parasitized histiocytes. Sections from mice after 1 or 7 days of *L(V)p* and *L(L)m* infection revealed a mild focal infiltrate of neutrophils in the deep dermis and the absence of parasites (not shown). By the 3rd week postinfection, a marked increase in the inflammatory infiltrate (granulocytic and histiocytic) was observed in the deep dermis of mice infected with both species. At this time, *L(L)m*-infected mice exhibited large infiltrates with formation of neutrophil microabscesses and the presence of abundant intracellular amastigotes, which contrasted with the smaller infiltrates, absence of microabscesses and scarce amastigotes observed in *L(V)p*-infected animals. The sections of week 7 postinfection showed a destructive massive inflammation induced by *L(L)m*, with heavily parasitized histiocytes dominating the infiltrate (also with the presence of granulocytes), and extensive zonal coagulative and liquefactive necrosis (which progressed from focal necrosis at earlier time points, not shown) with signs of vasculitis. Although *L(V)p*-infected footpads also increased the inflammatory infiltrate (histiocytic and granulocytic) and abundant intracellular amastigotes were evident at this time, a more benign histopathological picture was observed with no evidence of tissue destruction. Examination of *L(V)p*-infected foot at subsequent timepoints (11, 13, and 15 wpi) indicated no further growth of overall inflammation but progressive changes of composition towards a more lymphohistiocytic-dominated infiltrate with few granulocytes and less parasites compared with previous weeks (not shown). Weeks 13 and 15 of *L(V)p* infection were characterized by a lymphoplasmocytic-dominated infiltrate with the presence of less parasitized histiocytes (not shown). No collagen deposits or fibrosis were observed in all *L(V)p*-infected foot. These observations suggest the presence of a non-resolved chronic inflammatory process most likely maintained by persistent infection.

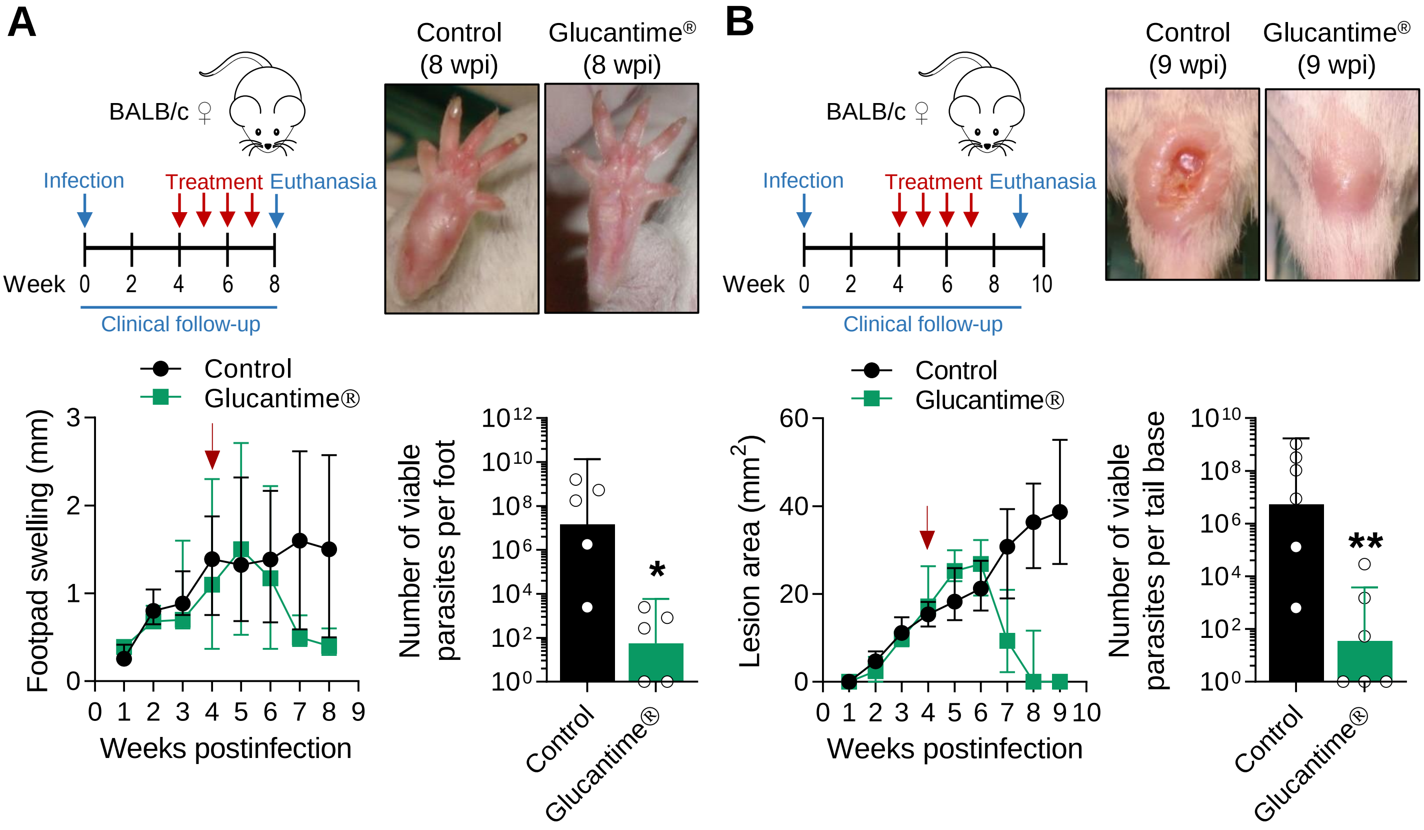

Supplementary Figure S12. The therapeutic effect of Glucantime® in BALB/c with established cutaneous lesions caused by infection with *L(V)p* in the footpad or the base of the tail. BALB/c mice were infected sc in the footpad **(A)** or the base of the tail **(B)** with 10^6 or 10^5 stationary promastigotes, respectively, and lesion development monitored weekly. Once lesions were established at the week 4 postinfection (red arrow in *lower panel*, *left*), mice were treated ip with Glucantime® (500 mg/kg, once a week, four weeks; as indicated by red arrows in the *upper panel*, *left*). The therapeutical effect was clinically confirmed by the decline in the footpad swelling **(A)** or size of the lesion **(B)** during the subsequent weeks, to reach a complete cure (*lower panel*, *left*). Representative photographs of treated and control mice at the week 8-9 postinfection are shown (*upper panel*, *right*). The burden of viable parasites at the site of injection (8th-9th week postinfection) indicated that clinical efficacy was the consequence of parasiticidal effects *in vivo* (approximately 5 log-fold reduction; *lower panel*, *right*). n=5-7. Data are shown as median ± interquartile range (for lesion size) or geometric mean ± 95%CI (for parasitic load). **p*<0.05, ***p*<0.01 (Mann-Whitney *U* test). wpi: weeks postinfection.

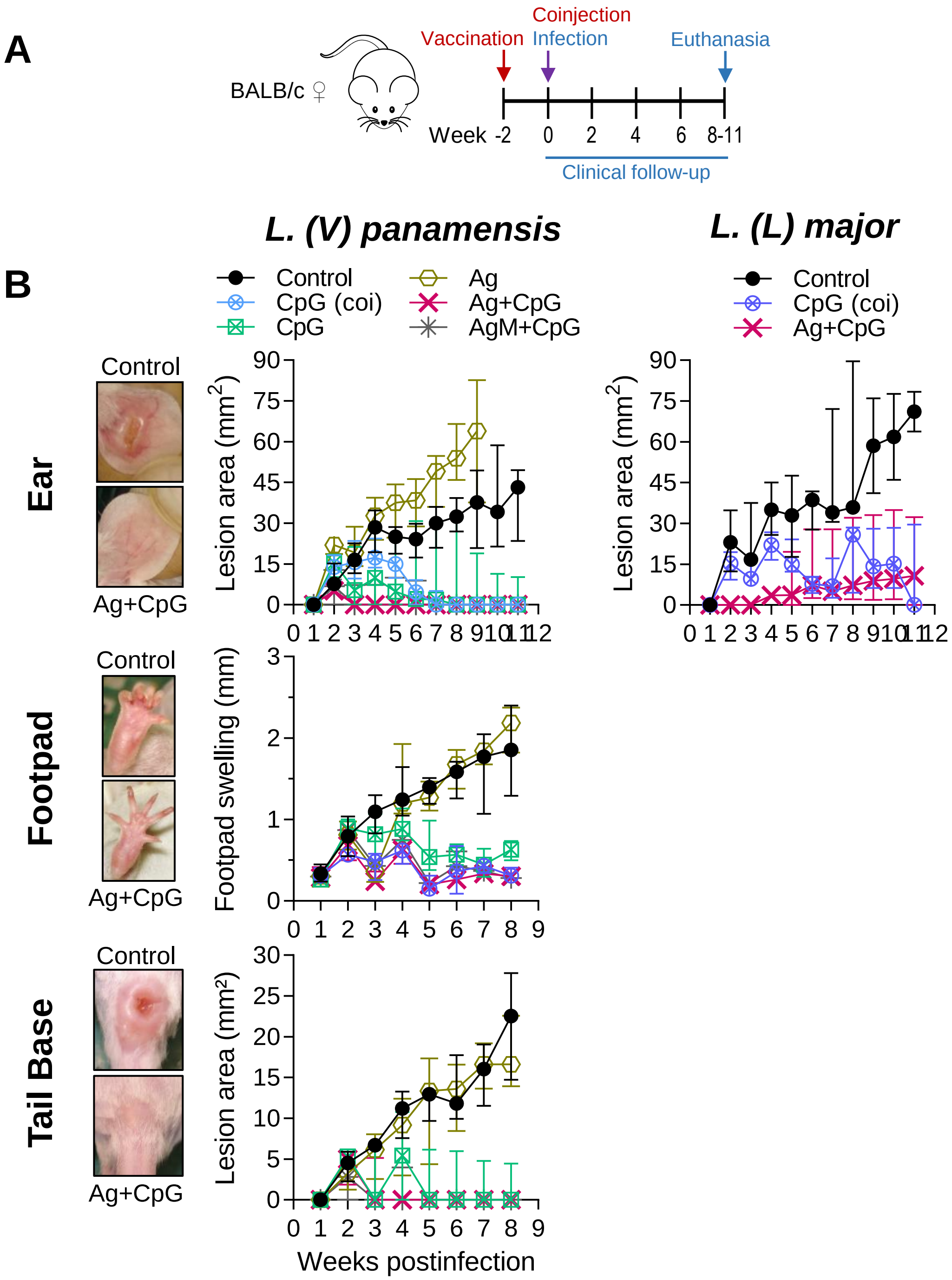

Supplementary Figure S13. A protective effect by CpG is also evidenced in the *L(V)p*-BALB/c mice model. BALB/c mice were used in experiments in which CpG (6.25 µg) was co-injected (coi) with the infective challenge or administered alone or in combination with Ag (12.5 µg) two weeks before **(A)**. The prophylactic effect of CpG alone or combined with Ag (total lysate) or membranous Ag (AgM, obtained by resuspending the pellet after centrifugating the total parasite lysate) was assessed in mice challenged in the ear, footpad or base of the tail with *L(V)p* **(A,B)**, as indicated. PBS- or Ag-injected mice were used as controls. The effect of CpG when co-delivered with the infective *L(V)p* challenge was also investigated in the same experiment. *L(L)m*-infected BALB/c mice, in which a protective effect of CpG has been demonstrated, were used as controls in a parallel experiment **(B,** *right***)**. Lesion development was monitored weekly and representative photograph of two of the experimental groups of *L(V)p*-infected animals are presented **(B,** *left***)**. All immunomodulatory interventions were performed at the same anatomical site of infection. Data are shown as median ± interquartile range (n=4-6 mice/group).

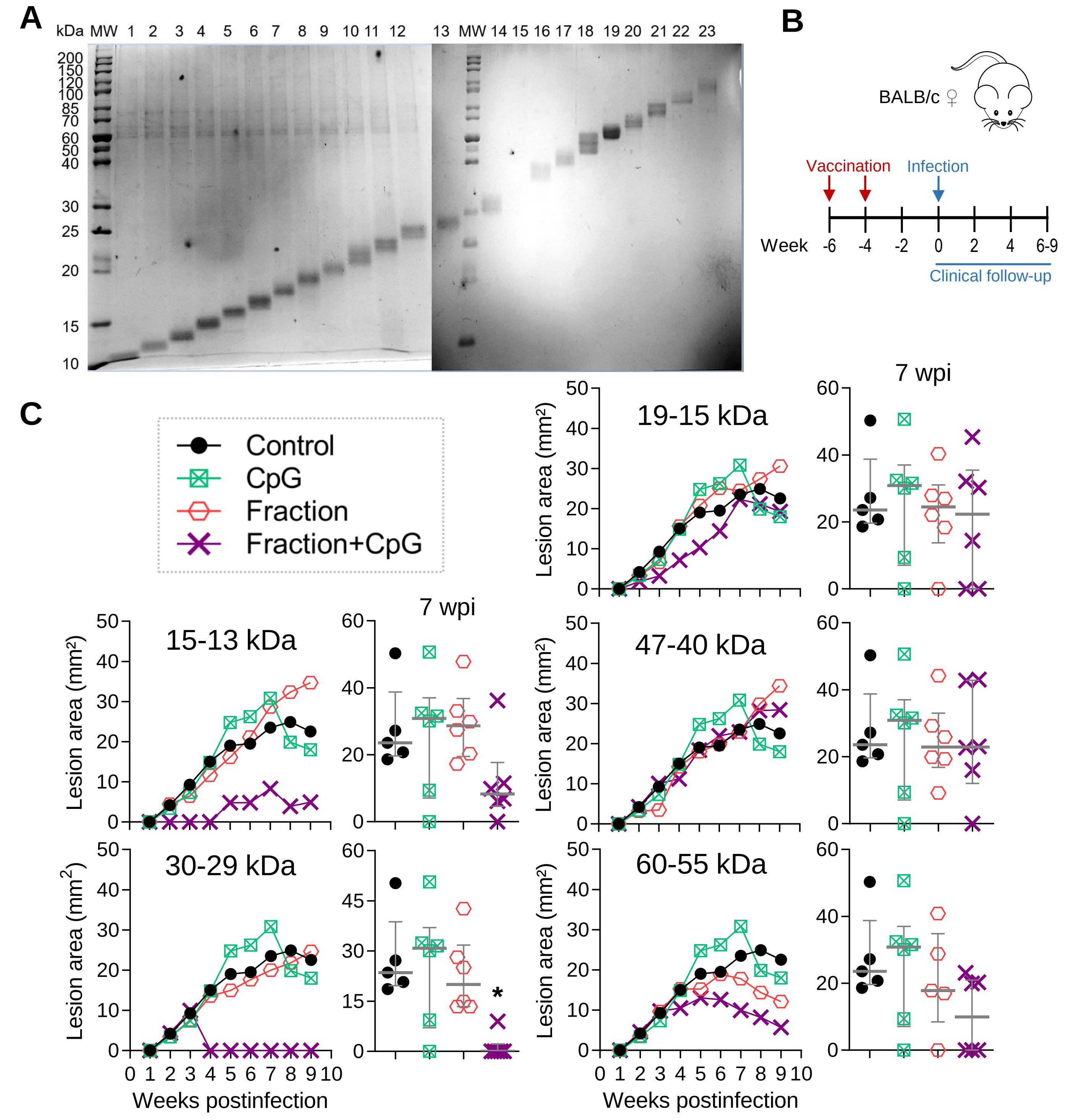

Supplementary Figure S14. The protective effect of parasite protein fractions obtained by electrophoresis/electroelution. Total promastigote lysate was separated by preparative electrophoresis and eluted from gels by electroelution as explained in **Supplementary Materials and Methods**. Silver-stained SDS/PAGE gels with the fractions obtained is shown **(A)**. Only 5 out of the 23 fractions obtained were sufficient for the *in vivo* testing. Mice were vaccinated id in the ear with 5 µg of the indicated protein fraction (the range of molecular weight in kDa is shown) alone or in combination with 2,5 µg CpG and boosted two weeks later with the same preparation to be infected with *L(V)p* four weeks after the boost, as indicated **(B)**. PBS- or CpG-injected mice served as negative controls. Lesion size was registered weekly and graphed **(C)** as the kinetics of lesion growth (*left*) or the size of the lesion in individual mice at the 7th week postinfection (*right*). Data are shown as the median or median ± interquartile range (n=5-6 mice/group). Note that the fraction 30-29 kDa induced complete protection of all the animals against infective challenge, when combined with CpG. *p<0.05 (Kruskal-Wallis test with Dunn's multiple comparison post-hoc test). MW: molecular weight. wpi: weeks postinfection.

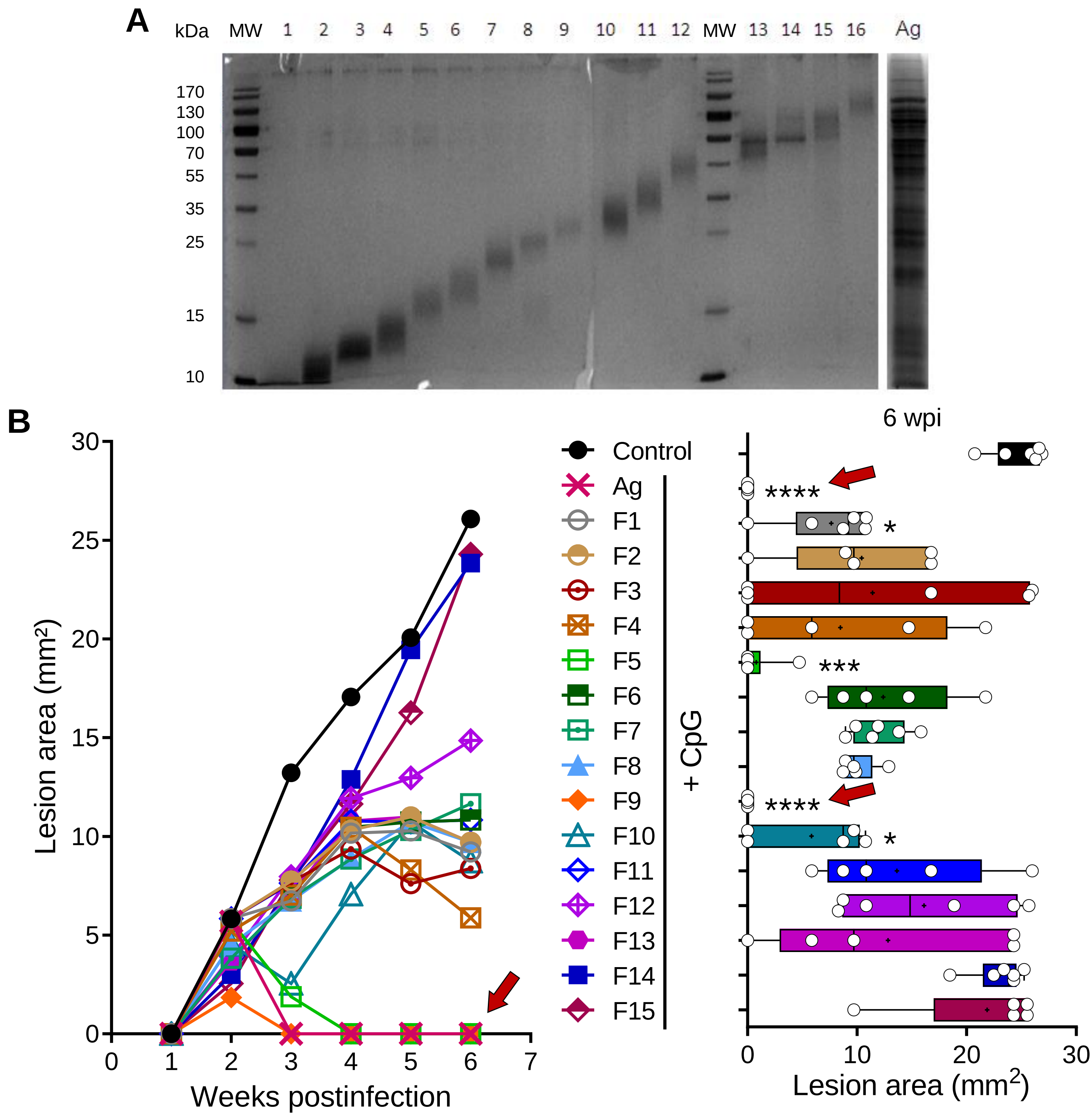

Supplementary Figure S15. The protective effect of parasite protein fractions obtained by electrophoresis and manual fractionation. Total promastigote lysate was separated by preparative electrophoresis and eluted from manually cut gel slices, as explained in **Supplementary Materials and Methods**. Silver-stained SDS/PAGE gels with the fractions obtained is shown **(A)**. Out of the 16 fractions, 15 were obtained in sufficient amounts for *in vivo* testing. Mice were vaccinated id in the ear with 6 µg of the indicated protein fraction in combination with 3.5 µg CpG and boosted two weeks later with the same preparation to be infected with *L(V)p* four weeks after the boost, as indicated in the **Supplementary Figure S14B**. PBS- or Ag+CpG-injected mice were used as negative and positive controls, respectively. Lesion size was registered weekly and graphed **(B)** as the kinetics of lesion growth (*left*) or the size of the lesion in individual mice at the 6th week postinfection (*right*). Data are shown as the median **(B**, *left***)** or using a "box and whiskers" plot **(B**, *right***)**. n=5-6 mice/group. Note that the fraction F9+CpG reproduced the protective effect of Ag+CpG (red arrow) and that the molecular weight range of F9 coincides with that of the protective fraction displayed in **Supplementary Figure S14**. MW: molecular weight. wpi: weeks postinfection. *p<0.05, ***p<0.001, ****p<0.0001 (Kruskal-Wallis test with Dunn's multiple comparison post-hoc test).

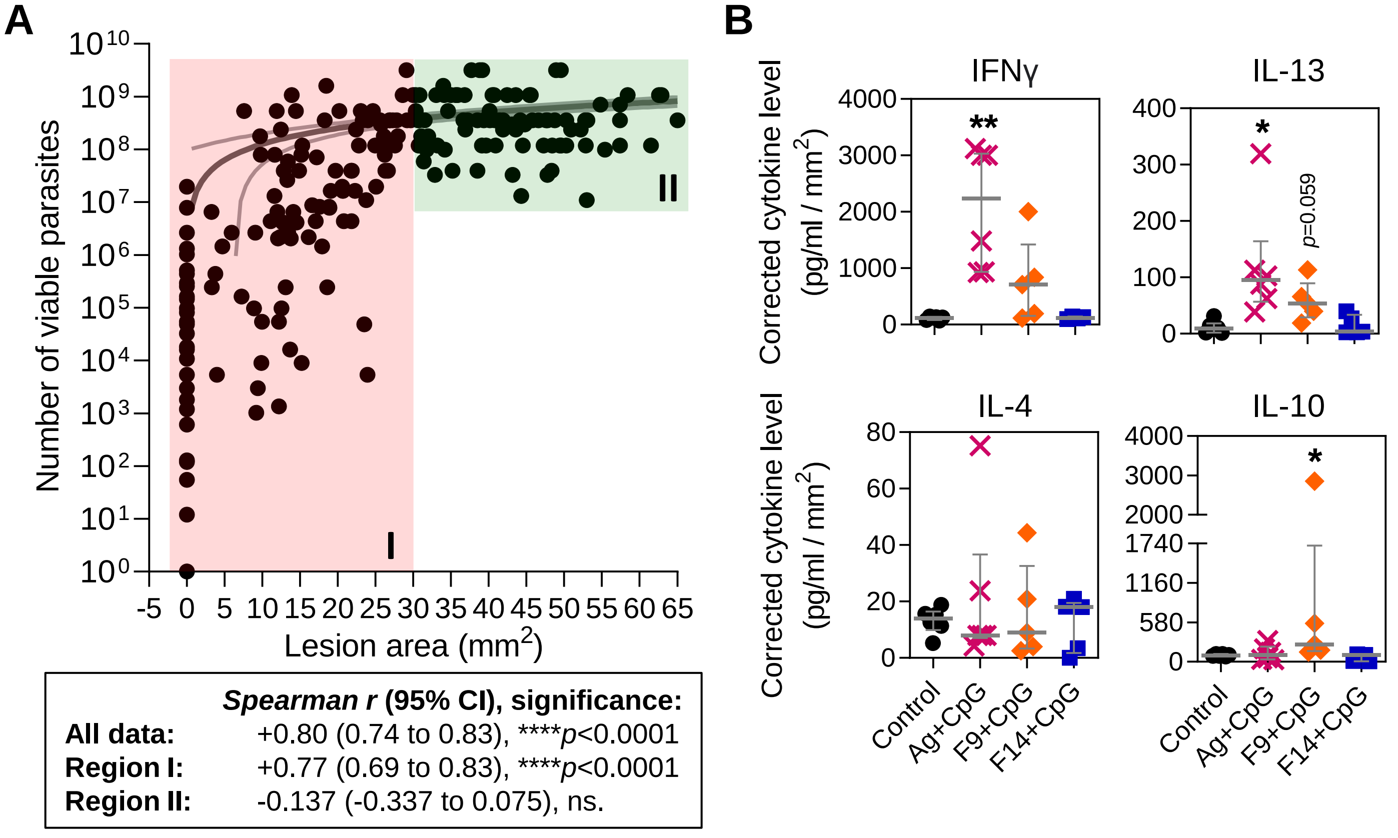

Supplementary Figure S16. Parasite burden correlates with lesion size in *L(V)p*-infected mice and allows cytokine level correction. Animals used in 14 independent experiments (n=260) for which individual paired data for ear lesion size and parasite load were available were pooled and plotted to perform a Spearman rank correlation analysis **(A).** Note the significant correlation observed when all data are included in the analysis or when only values below 30 mm^2 (which is the lesion size corresponding to the time in which maximal parasitic loads are reached in the kinetics presented in **Figure 1**) are considered (region I), but not when higher values (region II) are considered. Thus, lesion sizes of 30 mm^2 on could even double in size but remaining with a maximal parasitic load (10^8-10^9 viable parasites). Also note that ears could harbor significant parasite loads (up to 10^6-10^7 viable parasites) in the absence of measurable lesion. These observations confirm the presence of increase pathology under tight control of parasite multiplication, and latency, two typical characteristics of *L(V)p* infections in humans. The cytokine secretion analysis results displayed in **Figure 8G** were corrected by dividing the concentration of the indicated cytokine by the respective lesion size of individual mice **(B)**. Data of individual mice are shown, and the bars represent the median ± interquartile range (n=4-6 mice). Note that significant higher values of IFNγ and IL-13 associated with the protective effect of Ag+CpG, whereas increased IL-10 and a trend to higher IFNγ and IL-13 associated to F9+CpG-induced protection. Pattern of F14+CpG group looks more similar to the control group. *$p<0.05$, **$p<0.01$ (Kruskal-Wallis test with Dunn's multiple comparison post-hoc test).

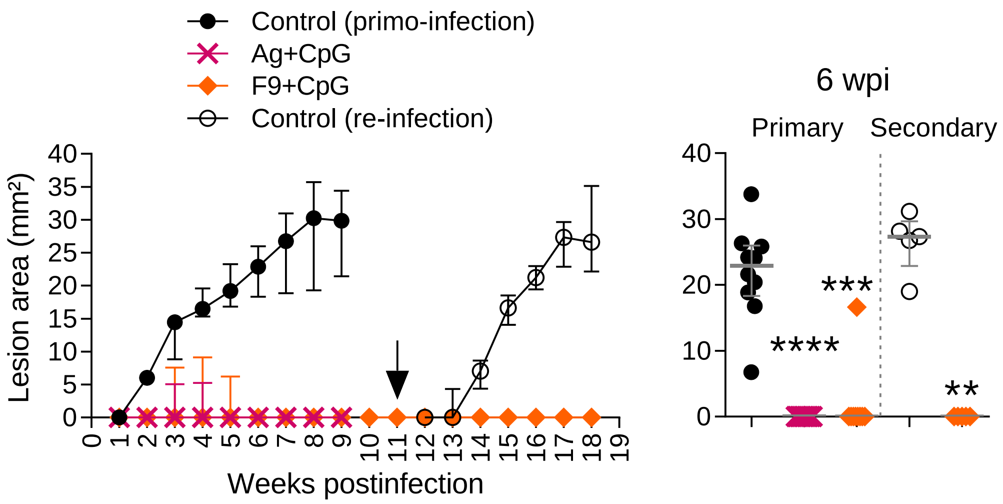

Supplementary Figure S17. F9+CpG protect mice from primary and secondary infection. BALB/c mice were vaccinated with F9+CpG or Ag+CpG or PBS (as control) and infected, in a similar manner of that shown in **Supplementary Figure S14B**. At the 11th week postinfection, F9+CpG-vaccinated mice received a secondary infection with 10^5 stationary *L(V)p* promastigotes in the contralateral ear (arrow) and monitored weekly. A group of age-matched naïve mice were also infected as controls of the infective secondary challenge, named "Control (re-infection)". Figures show the kinetics of lesion development (*left*) and the size of the lesions in individual mice at the 6th week postinfection (*right*). Data are shown as the median ± interquartile range (n=5-10 mice/group). In an independent experiment, also Ag+CpG-vaccinated mice resisted both primary and secondary *L(V)p* challenges (not shown). wpi: weeks postinfection. ***p*<0.01 (Mann-Whitney *U* test), ****p*<0.001, *****p*<0.001 (Kruskal-Wallis test with Dunn's multiple comparison post-hoc test).

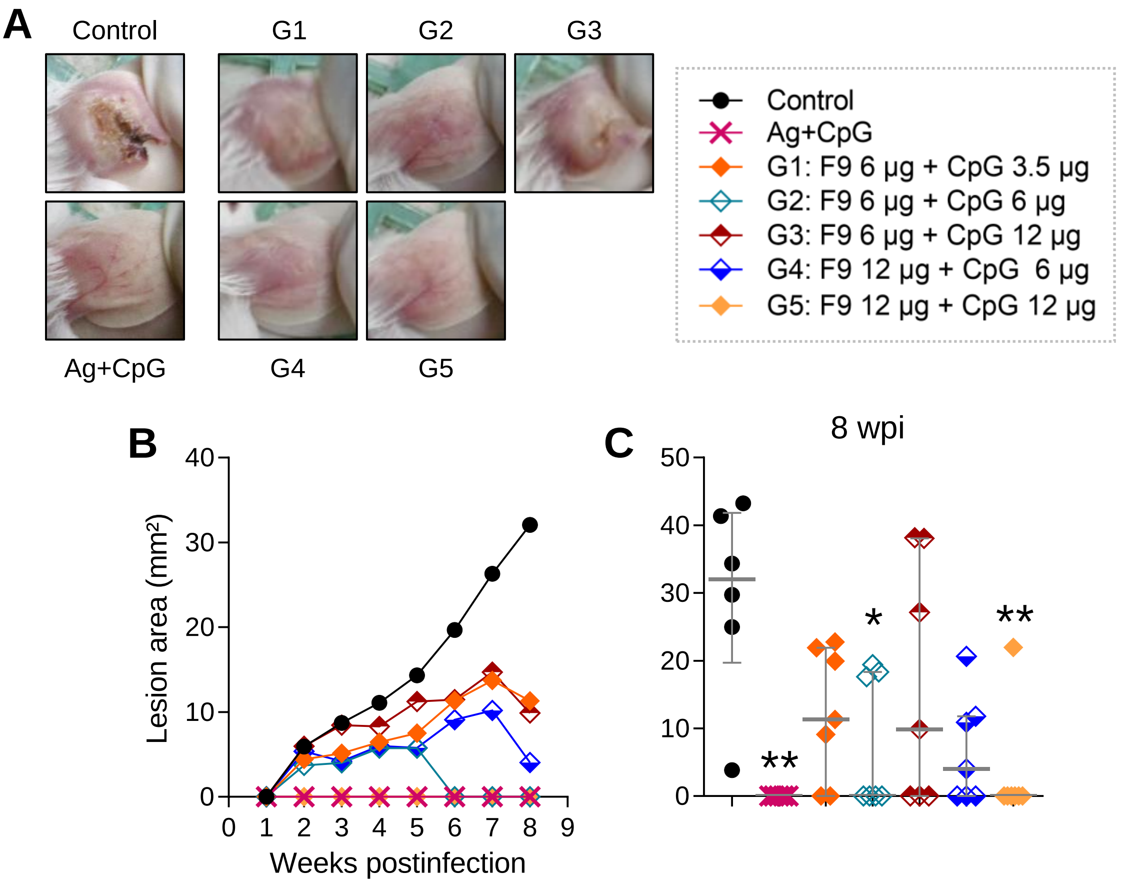

Supplementary Figure S18. Optimizing F9+CpG amounts in the vaccine formulation to maximize protection. BALB/c mice were vaccinated sc in the back with the indicated amounts of F9 protein fraction and CpG, boosted two weeks later with the same preparation, infected in the ears with *L(V)p* four weeks after the boost (as shown in **Supplementary Figure S14B)** and the lesion size registered weekly. PBS- or Ag+CpG-injected mice were used as negative and positive controls, respectively. Representative photographs illustrating the appearance of the ear lesions from each experimental group at the 8th week postinfection are shown **(A)**. The kinetics of lesion growth **(B)** or the size of the lesion in individual mice at the 8th week postinfection **(C)** are also shown. Data are presented as the median or median ± interquartile range (n=5-7 mice). Note that maximal level of protection is achieved with 12 µg/12 µg of F9/CpG in the vaccine formulation. wpi: weeks postinfection. **p*<0.05, ***p*<0.01 (Kruskal-Wallis test with Dunn's multiple comparison post-hoc test).
